# Association of Urinary EGF with Kidney Outcomes and Effects of Sodium-Glucose Cotransporter 2 Inhibition

**DOI:** 10.1681/ASN.0000001086

**Published:** 2026-04-02

**Authors:** Erik Moedt, Akihiko Koshino, Niels Jongs, Wenjun Ju, Viji Nair, Matthias Kretzler, Michael K. Hansen, Clare G. Arnott, David C. Wheeler, Peter Rossing, Steven G. Coca, Ann Hammarstedt, Peter J. Greasley, Petter Bjornstad, Stephan J.L. Bakker, Hiddo J.L. Heerspink

**Affiliations:** 1 Department of Clinical Pharmacy and Pharmacology, University Medical Center Groningen, University of Groningen, Groningen, The Netherlands; 2 Department of Nephrology and Rheumatology, Graduate School of Medical Sciences, Kanazawa University, Kanazawa, Japan; 3 Division of Nephrology, Department of Internal Medicine, University of Michigan, Ann Arbor, Michigan; 4 Janssen Research and Development, LLC, Spring House, Pennsylvania; 5 The George Institute for Global Health, Sydney, New South Wales, Australia; 6 Royal Prince Alfred Hospital, Camperdown, New South Wales, Australia; 7 Department of Renal Medicine, University College London, London, United Kingdom; 8 Steno Diabetes Center Copenhagen, Herlev, Denmark; 9 Icahn School of Medicine at Mount Sinai, New York, New York; 10 Late-Stage Development, Cardiovascular, Renal and Metabolism, BioPharmaceuticals R&D, AstraZeneca, Gothenburg, Sweden; 11 Research and Early Development, Cardiovascular, Renal and Metabolism, BioPharmaceuticals R&D, AstraZeneca, Gothenburg, Sweden; 12 Division of Metabolism, Endocrinology and Nutrition, Department of Medicine, University of Washington School of Medicine, Seattle, Washington; 13 Department of Internal Medicine, University Medical Center Groningen, University of Groningen, Groningen, The Netherlands

**Keywords:** biomarkers, CKD, diabetic kidney disease, EGF, SGLT2 inhibitors, tubule cells

## Abstract

**Key Points:**

- Lower urinary EGF levels, normalized to urinary creatinine, reflected impaired tubular health and were associated with higher kidney risk across CKD.
- Sodium-glucose cotransporter 2 inhibitors attenuated the decline in urinary EGF-creatinine ratio observed with placebo, supporting a role in maintaining tubular health.
- Early increases in urinary EGF-creatinine ratio were linked to lower kidney risk, adding prognostic value beyond albuminuria.

**Background:**

Urinary EGF is a marker of tubular repair capacity. Lower urinary EGF-creatinine ratio (uEGF/Cr) levels associate with kidney disease progression in patients with type 2 diabetes at early stages of CKD. In these patients, sodium-glucose cotransporter 2 inhibitors (SGLT2is) are associated with increased tubular EGF expression. In this study, we aimed to extend these findings to a broad CKD population with and without type 2 diabetes at various stages of CKD.

**Methods:**

We measured EGF in stored urine samples at baseline and year 1 in participants from the Canagliflozin Cardiovascular Assessment Study (CANVAS), Canagliflozin and Renal Events in Diabetes with Established Nephropathy Clinical Evaluation (CREDENCE), and Dapagliflozin and Prevention of Adverse Outcomes in CKD (DAPA-CKD) clinical trials. Associations of baseline and longitudinal uEGF/Cr with the composite kidney outcome (sustained ≥40% eGFR decline, kidney failure, or kidney-related death) were assessed using multivariable Cox regression, and associations with annual eGFR decline using a two-slope linear mixed-effects model. Treatment effects of SGLT2i on uEGF/Cr over time were analyzed with analysis of covariance.

**Results:**

In participants with type 2 diabetes from the CANVAS and CREDENCE trials (*N*=5978), higher baseline uEGF/Cr was associated with a lower risk of the composite kidney outcome (hazard ratio per two-fold higher uEGF/Cr, 0.87 [95% confidence interval, 0.80 to 0.94]). SGLT2i attenuated the decline in uEGF/Cr over 1 year compared with placebo by 6.7% (95% confidence interval, 2.5 to 10.8). Increases in uEGF/Cr from baseline to year 1 were independently associated with a lower risk of the kidney outcome, even after accounting for 1-year changes in albuminuria, eGFR, and other clinical variables. Replication analyses showed similar results in the DAPA-CKD trial (*N*=2450), with consistent findings in participants with and without diabetes.

**Conclusions:**

These results extend previous findings, supporting uEGF/Cr as a robust, independent biomarker of tubular health and kidney risk across diverse CKD populations. SGLT2i attenuated uEGF/Cr decline, and early changes in uEGF/Cr provided prognostic information beyond albuminuria.

## Visual Abstract

**Figure:**
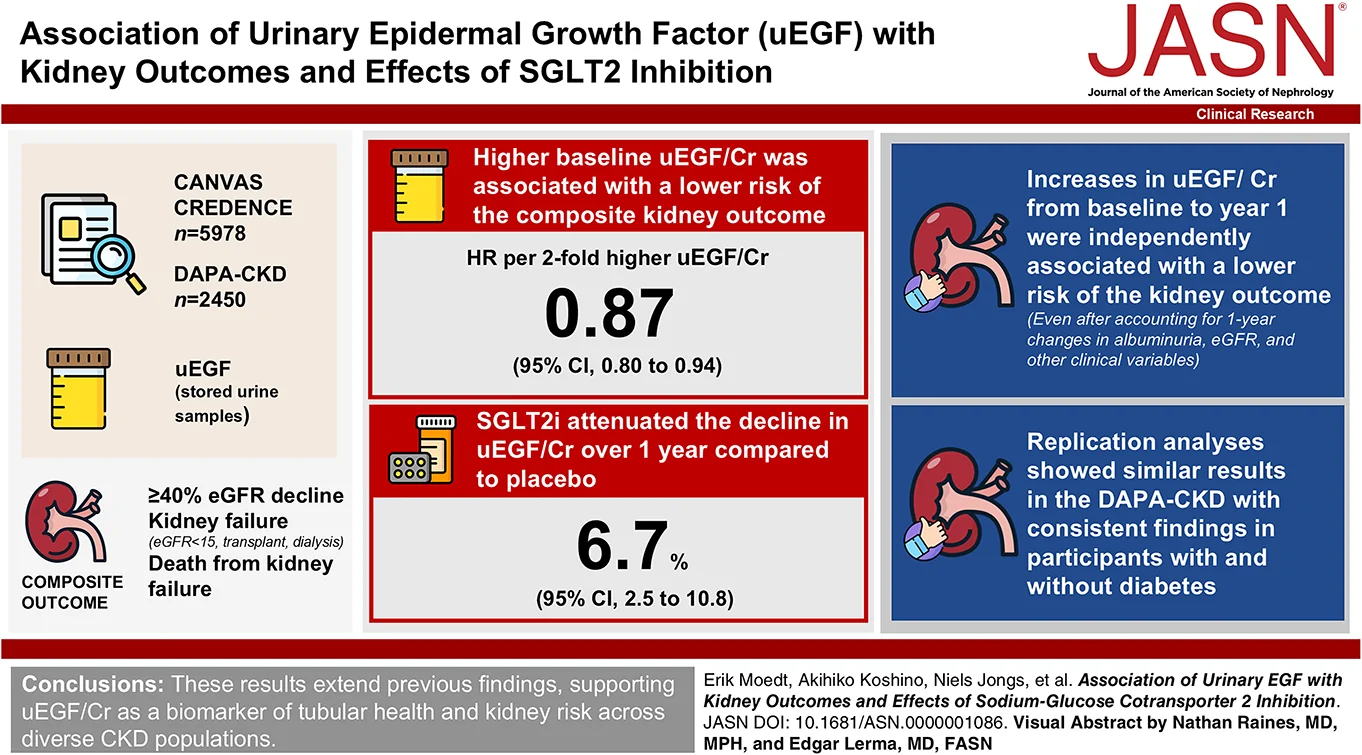

## Introduction

CKD is a progressive and incurable disease marked by high morbidity and mortality. Its prevalence is approximately 10% among the general adult population and elevated to 30%–40% among those with type 2 diabetes.^1^ Kidney function can be preserved effectively through pharmacologic interventions, especially when guided by pharmacodynamic biomarkers that enable monitoring of treatment efficacy.

EGF is an emerging biomarker that plays a pivotal role in tubular epithelial cell proliferation.^2,3^ It is primarily produced in the ascending loop of Henle and distal convoluted tubule and acts *via* binding to the EGF receptor, which is widely expressed in kidney tissue.^4,5^ Decreased urinary EGF (uEGF) excretion has been linked to increased kidney disease progression in patients with type 2 diabetes and is thought to be specific to kidney tubule damage and fibrosis.^6–9^

Sodium-glucose cotransporter 2 inhibitors (SGLT2is) reduce the risk of kidney disease progression in patients with type 2 diabetes.^10–12^ We previously demonstrated that the SGLT2i canagliflozin associates with higher tubular expression of EGF from research kidney biopsies and elevated levels of uEGF.^6^ In kidney tissue, it is involved in a downstream signaling cascade implicated in tubular repair and maintenance of epithelial integrity.^3^ Building on these findings, pathway analyses of kidney biopsies demonstrated that EGF plays a central role in the mechanism of action of SGLT2i in patients with type 2 diabetes.^6^ However, these results were primarily obtained from patients with preserved eGFR, low levels of albuminuria, and type 2 diabetes.

Accordingly, to extend these findings to a broader CKD population, we assessed the association between baseline uEGF normalized to urine creatinine (uEGF-creatinine ratio [uEGF/Cr]) and kidney outcomes in patients with type 2 diabetes across a wide range of CKD severity. We additionally evaluated its incremental predictive value beyond clinical risk factors, the relationship between baseline uEGF/Cr and annual eGFR decline, the effect of canagliflozin on uEGF/Cr over time, whether baseline uEGF/Cr modified SGLT2i treatment effects on kidney outcomes, and whether changes in uEGF/Cr from baseline to year 1 were associated with kidney outcomes using data from the Canagliflozin Cardiovascular Assessment Study (CANVAS) and Canagliflozin and Renal Events in Diabetes with Established Nephropathy Clinical Evaluation (CREDENCE) trials. Finally, we replicated these findings in an independent cohort of patients with CKD, with and without type 2 diabetes, treated with either dapagliflozin or placebo who participated in the Dapagliflozin and Prevention of Adverse Outcomes in CKD (DAPA-CKD) trial.

## Methods

### Patients and Study Design

To ensure a representative cohort of patients with type 2 diabetes across CKD stages, we combined the CANVAS and CREDENCE trials. CANVAS and CREDENCE were international, randomized, double-blinded, placebo-controlled trials assessing efficacy and safety of canagliflozin on cardiovascular and kidney outcomes in patients with type 2 diabetes. Detailed designs, inclusion criteria, and primary outcomes are published.^13,14^ CANVAS enrolled 4330 patients with type 2 diabetes, eGFR ≥30 ml/min per 1.73 m^2^, hemoglobin A1c (HbA1c) level ≥7.0% and ≤10.5%, either ≥30 years of age with a history of symptomatic atherosclerotic cardiovascular disease or ≥50 years of age with ≥2 cardiovascular risk factors, randomized to canagliflozin 100 or 300 mg orally once daily or placebo, followed for a median of 6.1 years (interquartile range [IQR], 5.8–6.4). CREDENCE enrolled 4401 patients with type 2 diabetes, eGFR ≥30 and <90 ml/min per 1.73 m^2^, urine albumin-creatinine ratio (UACR) >300 and ≤5000 mg/g, and HbA1c level ≥6.5% and ≤12.0%, randomized to canagliflozin 100 mg orally once daily or placebo, followed for a median of 2.6 years (IQR, 2.1–3.1).

For replication, we used data from DAPA-CKD, an international, double-blinded, randomized, placebo-controlled trial assessing efficacy and safety of dapagliflozin on cardiovascular and kidney outcomes in patients with CKD with and without type 2 diabetes. Detailed design, inclusion criteria, and primary outcomes are published.^15^ DAPA-CKD enrolled 4304 patients with eGFR ≥25 and <75 ml/min per 1.73 m^2^ and UACR >200 and ≤5000 mg/g, randomized to dapagliflozin 10 mg or placebo, followed for a median of 2.4 years (IQR, 2.0–2.7).

All participants provided written informed consent, and all participating centers obtained ethics approval for the protocol. Trials followed the Declaration of Helsinki and Good Clinical Practice guidelines and were registered with ClinicalTrials.gov (CANVAS: NCT01032629, CREDENCE: NCT02065791, and DAPA-CKD: NCT03036150).

### uEGF Assay

To quantify uEGF in available urine samples, we used the Mesoscale QuickPlex SQ 120 platform (Meso Scale Diagnostics, Rockville, MD), a high-performance electrochemiluminescence immunoassay. Samples were thawed, vortex-mixed, centrifuged, and underwent a single freeze-thaw cycle before measurement, which does not affect uEGF concentrations.^16^ Baseline and follow-up samples from each participant were analyzed on the same plate. In CANVAS, 6454 urine samples were assessed from April 2019 to February 2020, including 389 within-assay duplicate measurements, yielding a mean coefficient of variation (CV) of 5%. In CREDENCE, 5918 urine samples were assessed from March to September 2022, including 523 within-assay duplicate measurements, yielding a mean CV of 2%. In DAPA-CKD, 5112 urine samples were assessed from March to May 2025, including 454 within-assay duplicate measurements, yielding a mean CV of 3%.

### Kidney Outcomes

The composite kidney outcome was defined as a sustained ≥40% decline in eGFR from baseline, kidney failure (eGFR <15 ml/min per 1.73 m^2^, dialysis ≥28 days, or transplantation), or death due to kidney failure. Outcomes were adjudicated by a blinded end point adjudication committee using predetermined definitions.

### Statistical Analysis

Continuous variables with normal distributions were presented as means and SDs, skewed variables (UACR, uEGF, uEGF/Cr) as medians and IQRs, and categorical variables as frequencies and percentages. Geometric means were calculated to assess baseline uEGF/Cr differences across UACR and eGFR subgroups.

Associations between baseline uEGF/Cr and kidney outcome development were assessed using multivariable Cox proportional hazards regression, with hazard ratios (HRs) estimated per quartile and per two-fold higher. To examine the effect of covariates, two models were used. Model 1 was adjusted for age, sex, race, trial, and randomized treatment. Model 2 additionally included history of cardiovascular disease, HbA1c, smoking, systolic BP, body mass index, eGFR, and log-transformed UACR as covariates. All models adjusting for trial were limited to the combined CANVAS-CREDENCE dataset. Subgroup analysis using model 2 in CANVAS-CREDENCE examined associations across baseline UACR and eGFR, and within UACR subgroups using uEGF/Cr tertiles to assess risk gradients across albuminuria levels. Effect modification by treatment was evaluated using interaction terms and stratified analyses.

Incremental prognostic value was assessed by adding uEGF/Cr to a multivariable clinical model including all covariates from model 2. Discrimination was quantified using C-statistics with 95% confidence intervals (CIs) and the paired change (ΔC) obtained from 1000 bootstrap resamples. Model fit was compared with a likelihood ratio test.

Associations between uEGF/Cr and the annual eGFR slope decline were assessed using a two-slope linear mixed-effects model, including fixed effects for uEGF/Cr quartile, randomized treatment, a two-slope linear spline in follow-up time, all possible two- or three-way interactions, and the baseline covariates age, sex, race, trial, history of cardiovascular disease, systolic BP, HbA1c, eGFR, and log-transformed UACR.

Placebo-corrected effects of SGLT2i on uEGF/Cr were assessed with an analysis of covariance model, including the change in uEGF/Cr from baseline to year 1 as outcome and randomized treatment and log-transformed baseline uEGF/Cr as covariates. Only CANVAS-CREDENCE participants receiving canagliflozin 100 mg or placebo were included, excluding those who received 300 mg to harmonize treatment groups across trials. The treatment effect was assessed within different subgroups defined by baseline UACR, eGFR, and HbA1c.

Treatment effect of canagliflozin versus placebo on uEGF/Cr over time was estimated using a repeated measures model, adjusted for baseline uEGF/Cr. Owing to the availability of urine samples taken at year 3 in addition to baseline and year 1, only CREDENCE participants were included.

Treatment effects on the composite kidney outcome across baseline uEGF/Cr tertiles were evaluated using Cox proportional hazards regression models, adjusted for all covariates described above. Effect modification by baseline uEGF/Cr was assessed using interaction terms with treatment assignment. Absolute event rates were calculated per 100 person-years, and absolute risk reductions were derived from differences between placebo and SGLT2i. These analyses were restricted to the same CANVAS-CREDENCE participants receiving canagliflozin 100 mg or placebo.

The association of change in uEGF/Cr from baseline to year 1 with kidney outcome development was examined using a Cox proportional hazards regression model. A landmark approach was used, excluding any kidney outcome occurring before year 1. HRs were estimated for changes in uEGF/Cr categorized in quartiles and per two-fold higher. The second quartile was established as reference because this quartile included change in uEGF/Cr closest to 0 without including any increase in uEGF/Cr. Model 1 was adjusted for age, sex, race, trial, randomized treatment, and log-transformed uEGF/Cr at baseline. In model 2, we added baseline eGFR, log-transformed UACR, history of cardiovascular disease, HbA1c, systolic BP, body mass index, and changes in eGFR, UACR, systolic BP, and HbA1c from baseline to year 1.

Subgroup analyses were conducted using the fully adjusted model in CANVAS-CREDENCE to assess potential interaction by baseline UACR and eGFR. In addition, an evaluation of kidney outcome risk was performed across tertiles of change in UACR and change in uEGF/Cr from baseline to year 1, illustrating the interaction between these variables and their joint impact on kidney outcome risk. Participants in the second tertile of the change in UACR and uEGF/Cr were used as the reference group as these tertiles represent changes that were minimal or close to no change.

The results with *P* < 0.05 were considered statistically significant. All statistical analyses were conducted with R version 4.4.2 (R Project for Statistical Computing, http://www.r-project.org).

## Results

### Study Population

We included 5978 participants with available baseline urine samples: 3521 (81% of the complete cohort) from CANVAS and 2457 (56% of the complete cohort) from CREDENCE. Among these, 2713 (63%) CANVAS participants and 1967 (45%) CREDENCE participants also had urine samples available at year 1 after randomization. In CREDENCE, 682 (15%) participants had samples available at year 3 in addition to baseline and year 1. From DAPA-CKD, we included 2450 (57% of the complete cohort) with available baseline urine samples, of whom 2303 (54%) also had urine samples available at year 1.

Baseline characteristics for the CANVAS-CREDENCE and DAPA-CKD datasets are presented in Table 1. Characteristics were generally well-balanced between treatment arms (Supplemental Tables 1 and 2). In the CANVAS-CREDENCE dataset, the baseline median uEGF was 2.93 ng/ml (IQR, 1.39–5.71) and the median uEGF/Cr was 3.69 ng/mg (IQR, 2.08–6.25). uEGF and uEGF/Cr were higher in CANVAS compared with CREDENCE (Supplemental Table 1). In DAPA-CKD, the baseline median uEGF and uEGF/Cr were markedly lower at 0.99 ng/ml (IQR, 0.54–1.76) and 1.32 ng/mg (IQR, 0.85–2.14), respectively.

**Table 1:** Baseline characteristics of the total population for the CANVAS-CREDENCE dataset and DAPA-CKD participants

Characteristic	CANVAS-CREDENCE	DAPA-CKD
	*N*=5978	*N*=2450
Age, yr	63 (8)	62 (12)
Male sex, *n* (%)	3986 (67)	1636 (67)
**Race, *n* (%)**		
Asian	676 (11)	536 (22)
Black	195 (3)	92 (4)
Other	477 (8)	285 (12)
White	4630 (77)	1537 (63)
Smoking, *n* (%)	1007 (17)	332 (14)
Diabetes, *n* (%)	5978 (100)	1693 (69)
History of cardiovascular disease, *n* (%)	3362 (56)	973 (40)
BMI, kg/m^2^	32.3 (6.2)	30.3 (6.3)
Systolic BP, mm Hg	138 (16)	138 (17)
HbA1c, %	8.2 (1.1)	7.1 (1.7)
eGFR, ml/min per 1.73 m^2^	69 (21)	44 (13)
UACR, mg/g (IQR)	70.7 (9.30–741)	942 (462–1896)
uEGF, ng/ml (IQR)	2.93 (1.39–5.71)	0.99 (0.54–1.76)
uEGF/Cr, ng/mg (IQR)	3.69 (2.08–6.25)	1.32 (0.85–2.14)

Numerical variables with a normal distribution are presented as mean (SD). Variables with a skewed distribution, urine albumin-creatinine ratio, urinary EGF, and urinary EGF-creatinine ratio, are reported as median (interquartile range). Categorical variables are presented as frequency (%). BMI, body mass index; CANVAS, Canagliflozin Cardiovascular Assessment Study; CREDENCE, Canagliflozin and Renal Events in Diabetes with Established Nephropathy Clinical Evaluation; DAPA-CKD, Dapagliflozin and Prevention of Adverse Outcomes in CKD; HbA1c, hemoglobin A1c; IQR, interquartile range; UACR, urine albumin-creatinine ratio; uEGF, urinary EGF; uEGF/Cr, urinary EGF-creatinine ratio.

At baseline, uEGF/Cr varied across UACR and eGFR subgroups, with higher concentrations observed in progressively higher eGFR subgroups and lower UACR subgroups (Table 2). For example, participants in the highest eGFR (>90 ml/min per 1.73 m^2^) and lowest UACR (<30 mg/g) subgroup had a geometric mean uEGF/Cr of 6.80 ng/mg (95% CI, 6.34 to 7.29), compared with a median of 1.67 ng/mg (95% CI, 1.59 to 1.76) in participants in the lowest eGFR (30–60 ml/min per 1.73 m^2^) and highest UACR (>1000 mg/g) subgroup. In DAPA-CKD, the geometric mean uEGF/Cr ranged from 3.31 ng/mg (95% CI, 2.71 to 4.06) in the highest eGFR (61–90 ml/min per 1.73 m^2^) and lowest UACR (30–300 mg/g) subgroup to 1.23 ng/mg (95% CI, 1.18 to 1.29) in the lowest eGFR (30–60 ml/min per 1.73 m^2^) and highest UACR (>1000 mg/g) subgroup.

**Table 2:** Geometric mean (95% confidence interval) of urinary EGF-creatinine ratio (ng/mg) by baseline eGFR and urine albumin-creatinine ratio subgroups

Subgroup	CANVAS-CREDENCE			DAPA-CKD	
	eGFR 30–60 ml/min per 1.73 m^2^	eGFR 61–90 ml/min per 1.73 m^2^	eGFR >90 ml/min per 1.73 m^2^	eGFR 30–60 ml/min per 1.73 m^2^	eGFR 61–90 ml/min per 1.73 m^2^
UACR <30 mg/g	3.8 (3.5 to 4.1)	5.3 (5.1 to 5.5)	6.8 (6.3 to 7.3)	1.4 (1.4 to 1.4)	—
UACR 30–300 mg/g	2.5 (2.3 to 2.7)	4.3 (4.0 to 4.6)	6.0 (5.4 to 6.6)	1.7 (1.6 to 1.8)	3.3 (2.7 to 4.1)
UACR 301–1000 mg/g	2.0 (1.9 to 2.1)	3.1 (2.9 to 3.3)	4.3 (3.5 to 5.2)	1.4 (1.4 to 1.5)	2.9 (2.6 to 3.2)
UACR >1000 mg/g	1.7 (1.6 to 1.8)	2.7 (2.5 to 2.9)	4.3 (3.6 to 5.1)	1.2 (1.2 to 1.3)	2.2 (1.9 to 2.4)

CANVAS, Canagliflozin Cardiovascular Assessment Study; CREDENCE, Canagliflozin and Renal Events in Diabetes with Established Nephropathy Clinical Evaluation; DAPA-CKD, Dapagliflozin and Prevention of Adverse Outcomes in CKD; UACR, urine albumin-creatinine ratio.

### Association between uEGF/Cr at Baseline and Kidney Outcome

Over a median follow-up of 5.0 years (IQR, 2.6–6.1), 430 (7%) CANVAS-CREDENCE participants experienced the kidney outcome. Baseline uEGF/Cr demonstrated a significant association with the kidney outcome, with a corresponding HR per two-fold higher uEGF/Cr of 0.87 (95% CI, 0.80 to 0.94; Figure 1) in the fully adjusted model. Participants in the highest quartile of uEGF/Cr had a statistically significant almost two-fold lower risk (HR, 0.57; 95% CI, 0.38 to 0.85) compared with those in the lowest quartile.

**Figure 1:**
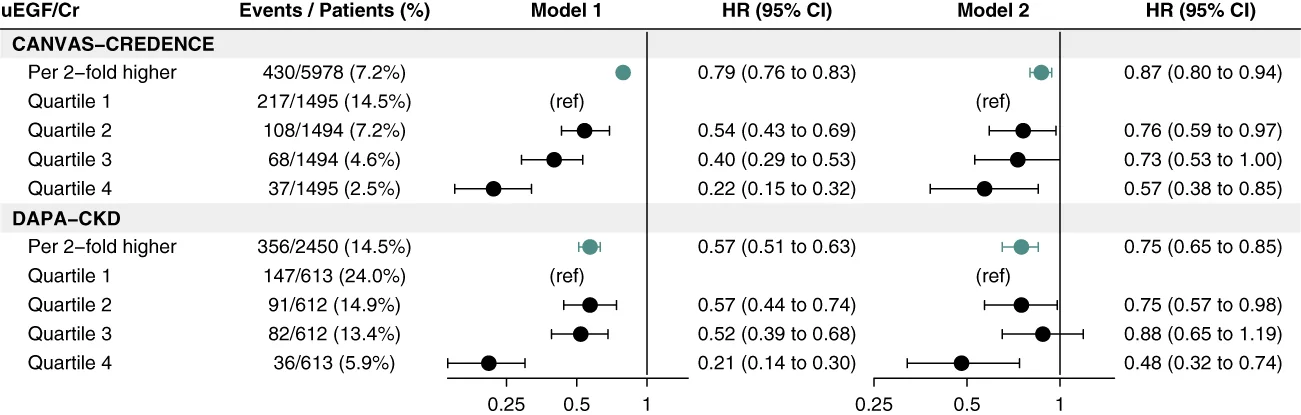
**Associations of baseline uEGF/Cr with composite kidney outcome (40% eGFR decline/kidney failure/kidney death).** Association is shown between uEGF/Cr with the kidney outcome when uEGF/Cr is modeled as a categorical (quartiles) and as a continuous (per two-fold higher) variable. Models are adjusted for the following covariates: model 1: age, sex, race, trial (not for DAPA-CKD), and randomized treatment. Model 2: model 1 covariates+history of cardiovascular disease, HbA1c, smoking, systolic BP, BMI, eGFR, and log-transformed UACR. In CANVAS-CREDENCE, uEGF/Cr quartile ranges for Q1–Q4 were 0.00009–2.08, 2.08–3.69, 3.70–6.25, and 6.25–163.4 ng/mg, respectively. In DAPA-CKD, uEGF/Cr quartile ranges for Q1–Q4 were 0.056–0.85, 0.85–1.32, 1.32–2.14, and 2.14–23.0 ng/mg, respectively. BMI, body mass index; CANVAS, Canagliflozin Cardiovascular Assessment Study; CI, confidence interval; CREDENCE, Canagliflozin and Renal Events in Diabetes with Established Nephropathy Clinical Evaluation; DAPA-CKD, Dapagliflozin and Prevention of Adverse Outcomes in CKD; HbA1c, hemoglobin A1c; HR, hazard ratio; UACR, urine albumin-creatinine ratio; uEGF/Cr, urinary EGF-creatinine ratio.

No significant effect modification was observed across UACR (*P* for interaction = 0.79) or eGFR subgroups (*P* for interaction = 0.23; Supplemental Figure 1). Higher uEGF/Cr tertiles were consistently associated with a lower risk of kidney outcomes across all UACR subgroups (Figure 2A).

**Figure 2:**
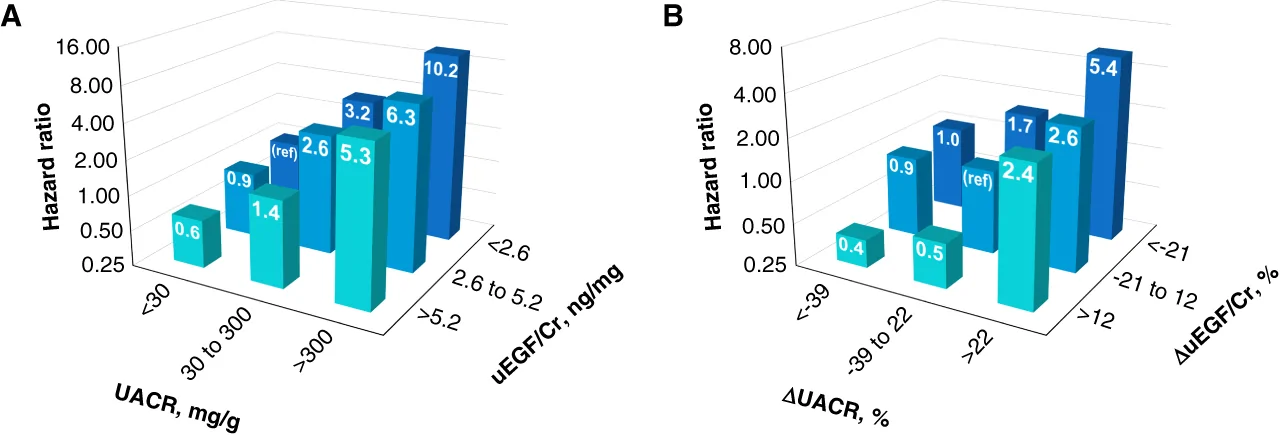
**Associations of baseline and 1-year changes in uEGF/Cr with composite kidney outcome across uEGF/Cr and urine albumin-creatinine ratio subgroups in CANVAS-CREDENCE.** Associations are shown between (A) baseline uEGF/Cr and the kidney outcome by UACR subgroups and uEGF/Cr tertiles and (B) change in uEGF/Cr from baseline to year 1 and the kidney outcome by tertiles of change in UACR and uEGF/Cr. HRs are expressed (A) per two-fold higher uEGF/Cr and adjusted for age, sex, randomized treatment, trial, history of cardiovascular disease, HbA1c, smoking, systolic BP, BMI, and eGFR, and (B) per two-fold increase in uEGF/Cr and adjusted for baseline log uEGF/Cr, age, sex, randomized treatment, trial, history of cardiovascular disease, HbA1c, smoking, systolic BP, BMI, and eGFR, and change in eGFR, systolic BP, and HbA1c from baseline to year 1.

Adding uEGF/Cr to an exhaustive multivariable clinical model increased the C-statistic from 0.859 (95% CI, 0.840 to 0.877) to 0.861 (95% CI, 0.842 to 0.879), corresponding to a ΔC of 0.002 (95% CI, 0.0004 to 0.0039). Model fit was significantly improved compared with the clinical model (likelihood ratio test *P* = 0.001).

### Association between uEGF/Cr and Annual eGFR Slope Decline

Analysis of the association between uEGF/Cr at baseline and annual eGFR slope decline from baseline to week 130 demonstrated a consistent steeper eGFR decline in CANVAS-CREDENCE participants with lower baseline uEGF/Cr. Notably, those in the lowest quartile experienced a mean annual eGFR slope decline of −4.04 ml/min per 1.73 m^2^ (SEM, 0.15), compared with −1.95 ml/min per 1.73 m^2^ (SEM, 0.16) in the highest quartile (Figure 3).

**Figure 3:**
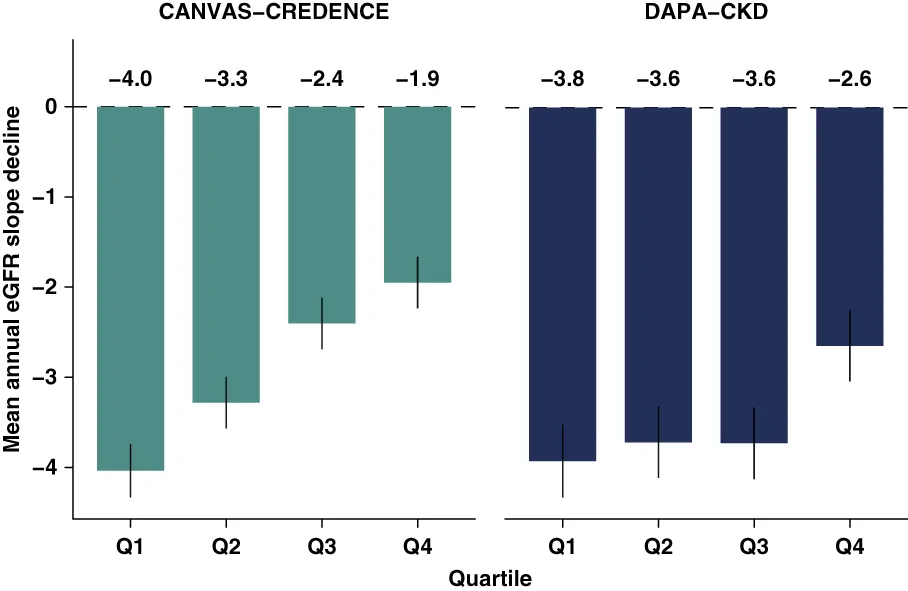
**Association between baseline uEGF/Cr and mean annual eGFR slope decline.** Association is shown between uEGF/Cr and annual eGFR slope decline when uEGF/Cr is modeled as a categorical (quartiles) variable. The model included fixed effects for uEGF/Cr quartile, randomized treatment, a two-slope linear spline in follow-up time, all possible two- or three-way interactions, and baseline covariates: age, sex, race, trial (not for DAPA-CKD), history of cardiovascular disease, systolic BP, HbA1c, eGFR, and log-transformed UACR. In CANVAS-CREDENCE, uEGF/Cr quartile ranges for Q1–Q4 were 0.00009–2.08, 2.08–3.69, 3.69–6.25, and 6.25–163.4 ng/mg, respectively. In DAPA-CKD, uEGF/Cr quartile ranges for Q1–Q4 were 0.056–0.85, 0.85–1.32, 1.32–2.14, and 2.14–23.0 ng/mg, respectively.

### Effect of Canagliflozin on uEGF/Cr

Among 3766 CANVAS-CREDENCE participants randomized to canagliflozin 100 mg, canagliflozin significantly attenuated the reduction in uEGF/Cr observed in the placebo group from baseline to year 1 by 6.7% (95% CI, 2.5 to 10.8; Table 3). This effect was consistent across subgroups defined by UACR (*P* for interaction = 0.98), eGFR (*P* for interaction = 0.15), and HbA1c (*P* for interaction = 0.55). The SDs of log-transformed change in uEGF/Cr were 0.76 (placebo) and 0.68 (canagliflozin).

**Table 3:** Effect of 1-year sodium-glucose cotransporter 2 inhibitor (canagliflozin 100 mg or dapagliflozin 10 mg) treatment compared with placebo on urinary EGF-creatinine ratio

Subgroup	Geometric Mean Baseline uEGF/Cr, ng/mg		Change from Baseline at Year 1, %			
	SGLT2i	Placebo	SGLT2i (95% CI)	Placebo (95% CI)	Between-Group Difference (95% CI)	*P* for Interaction
**CANVAS-CREDENCE**	3.3	3.3	−4.5 (−7.4 to −1.6)	−11.1 (−14.1 to −8.1)	6.7 (2.5 to 10.8)	
UACR, mg/g						0.98
*<30*	5.7	5.5	−3.1 (−8.1 to 2.0)	−10.5 (−15.7 to −5.3)	7.5 (0.2 to 14.7)	
*30–300*	3.6	3.0	0.0 (−5.7 to 5.7)	−4.8 (−11.0 to 1.5)	4.7 (−3.7 to 13.2)	
*301–1000*	2.4	2.5	−3.4 (−8.8 to 1.9)	−9.5 (−14.9 to −4.1)	6.1 (−1.5 to 13.7)	
*>1000*	2.0	2.1	−10.5 (−16.1 to −5.0)	−17.7 (−23.5 to −12.0)	7.2 (−0.8 to 15.2)	
eGFR, ml/min per 1.73 m^2^						0.15
*30–60*	2.0	2.2	−7.1 (−12.1 to −2.1)	−14.3 (−19.5 to −9.2)	7.2 (0.1 to 14.4)	
*61–90*	4.2	4.1	−4.7 (−8.5 to −0.9)	−9.9 (−13.8 to −5.9)	5.2 (−0.3 to 10.6)	
*>90*	6.3	5.9	1.8 (−4.6 to 8.3)	−1.9 (−8.6 to 4.9)	3.7 (−5.7 to 13.1)	
HbA1c, %						0.55
*≤8.0*	3.3	3.4	−2.8 (−6.7 to 1.1)	−10.8 (−14.7 to −6.9)	8.0 (2.5 to 13.5)	
*>8.0*	3.4	3.3	−6.1 (−10.4 to −1.9)	−11.6 (−16.1 to −7.0)	5.4 (−0.8 to 11.7)	
**DAPA-CKD**	1.4	1.3	−8.3 (−11.6 to −5.0)	−17.8 (−21.2 to −14.5)	9.5 (4.9 to 14.2)	
UACR, mg/g						0.07
*30–300*	1.8	1.6	−6.2 (−12.9 to 0.5)	−5.0 (−12.6 to 2.6)	−1.2 (−11.4 to 9.0)	
*301–1000*	1.5	1.5	−6.3 (−11.9 to −0.7)	−12.9 (−18.4 to −7.4)	6.6 (−1.3 to 14.4)	
*>1000*	1.2	1.2	−10.4 (−14.9 to −5.8)	−24.9 (−29.5 to −20.2)	14.5 (8.0 to 21.0)	
eGFR, ml/min per 1.73 m^2^						0.86
*30–60*	1.4	1.4	−7.2 (−10.8 to −3.7)	−15.7 (−19.3 to −12.1)	8.5 (3.4 to 13.5)	
*61–90*	2.7	2.5	−6.3 (−15.5 to 2.9)	−17.6 (−27.7 to −7.6)	11.3 (−2.3 to 25.0)	
HbA1c, %						0.23
*<6.5*	1.3	1.3	−13.4 (−18.7 to −8.1)	−18.8 (−24.1 to −13.5)	5.4 (−2.1 to 12.9)	
*6.5–8.0*	1.4	1.3	−8.4 (−14.3 to −2.5)	−19.0 (−25.3 to −12.8)	10.6 (2.0 to 19.3)	
*>8.0*	1.5	1.5	0.6 (−4.9 to 6.0)	−14.9 (−20.4 to −9.4)	15.5 (7.7 to 23.2)	

Treatment effect is shown for the overall population and in subgroups defined by baseline urine albumin-creatinine ratio, eGFR, and hemoglobin A1c. The model was adjusted for urinary EGF-creatinine ratio at baseline. CANVAS, Canagliflozin Cardiovascular Assessment Study; CI, confidence interval; CREDENCE, Canagliflozin and Renal Events in Diabetes with Established Nephropathy Clinical Evaluation; DAPA-CKD, Dapagliflozin and Prevention of Adverse Outcomes in CKD; HbA1c, hemoglobin A1c; SGLT2i, sodium-glucose cotransporter 2 inhibitor; UACR, urine albumin-creatinine ratio; uEGF/Cr, urinary EGF-creatinine ratio.

In a subset of 2038 patients from the CREDENCE trial with available longitudinal data, canagliflozin attenuated the uEGF/Cr decline by 9.8% (95% CI, 4.4 to 15.4) over time, with a difference of 8.1% (95% CI, 1.1 to 15.6) at year 1 and a 16.5% (95% CI, 2.1 to 32.8) at year 3 compared with placebo (Supplemental Table 3).

### Effect of Canagliflozin on Composite Kidney Outcomes by Baseline uEGF/Cr

Canagliflozin 100 mg versus placebo reduced the risk of the composite kidney outcome by 45% (HR, 0.55; 95% CI, 0.45 to 0.67; Supplemental Table 4). This relative treatment effect was consistent across baseline uEGF/Cr tertiles (*P* for interaction = 0.78). Absolute risk reductions varied numerically across tertiles, with the largest reductions observed in the lowest tertile.

### Association of 1-Year Change in uEGF/Cr with Kidney Outcome

Among 4639 CANVAS-CREDENCE participants with available urine samples at baseline and year 1, the median uEGF/Cr was 3.76 ng/mg (IQR, 2.15–6.39). After 1 year, 312 (7%) experienced the kidney outcome. Each two-fold increase in uEGF/Cr from baseline to year 1 was associated with a HR of 0.75 (95% CI, 0.69 to 0.81; Figure 4) in the fully adjusted model. Participants in the highest quartile of uEGF/Cr change had a statistically significant two-fold lower risk for the kidney outcome compared with those in the lowest quartile.

**Figure 4:**
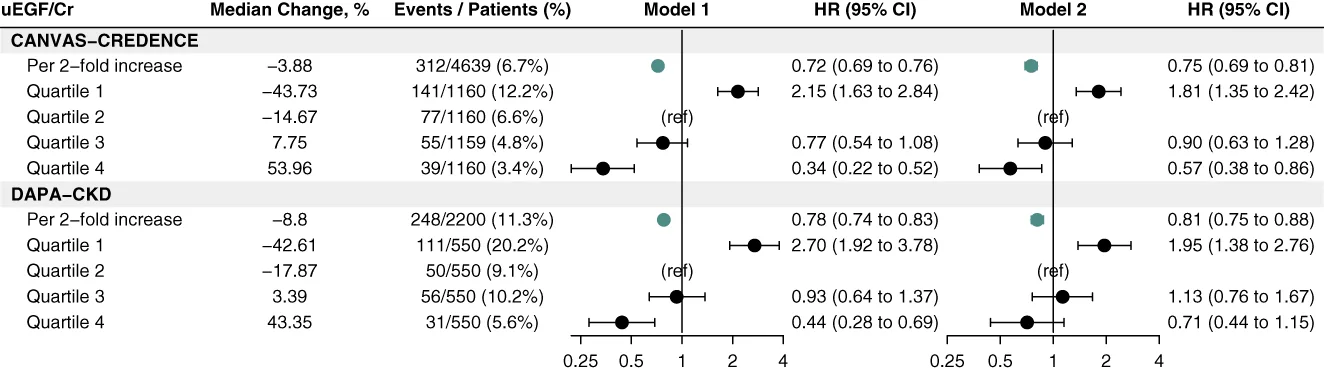
**Associations of change in uEGF/Cr from baseline to year 1 with composite kidney outcome.** Association is shown between change in uEGF/Cr from baseline to year 1 with the kidney outcome when uEGF/Cr change is modeled as a categorical (quartiles) and as a continuous (per two-fold increase) variable. Models are adjusted for the following covariates: model 1: baseline log uEGF/Cr, age, sex, race, trial (not for DAPA-CKD), and randomized treatment. Model 2: model 1 covariates+history of cardiovascular disease, HbA1c, systolic BP, BMI, eGFR, and log UACR at baseline and change in eGFR, UACR, systolic BP, and HbA1c from baseline to year 1. In CANVAS-CREDENCE, uEGF/Cr change quartile ranges for Q1–Q4 were −993.6 to −31.5, −31.5 to −3.96, −3.94% to 20.7%, and 20.7% to 1110.8%, respectively. In DAPA-CKD, uEGF/Cr change quartile ranges for Q1–Q4 were −973.2 to −35.7, −35.6 to −9.23, −9.20% to 16.6%, and 16.7%–226.9%, respectively.

No significant interaction was seen across eGFR subgroups (*P* for interaction = 0.47; Supplemental Figure 2). A significant interaction was found for UACR subgroups (*P* for interaction = 0.02), with a stronger inverse association between uEGF/Cr change and kidney outcomes in participants with UACR >300 mg/g compared with those with lower albuminuria levels. No interaction by treatment allocation was observed (*P* for interaction = 0.71).

When uEGF/Cr was jointly analyzed with the 1-year change in UACR, the associations between increased uEGF/Cr and lower kidney risk persisted across all tertiles of change in UACR from baseline to year 1 (Figure 2B), suggesting independent contribution of both biomarkers to risk stratification.

### Replication in DAPA-CKD

To replicate findings from the CANVAS-CREDENCE dataset, uEGF was measured in DAPA-CKD. Here, uEGF/Cr was similarly associated with the kidney outcomes, with a HR per two-fold higher of 0.75 (95% CI, 0.65 to 0.85; Figure 1). The addition of uEGF/Cr to a clinical model increased the C-statistic from 0.785 (95% CI, 0.755 to 0.811) to 0.790 (95% CI, 0.761 to 0.815; ΔC, 0.005; 95% CI, −0.0003 to 0.0104), and model fit was significantly improved (likelihood ratio test *P* < 0.001). Parallel to CANVAS-CREDENCE, the relative treatment effect of dapagliflozin on the composite kidney outcome was consistent across baseline uEGF/Cr tertiles, with numerically larger absolute risk reductions observed in the lowest tertile (Supplemental Table 3). After 1 year, dapagliflozin increased uEGF/Cr by 9.5% (95% CI, 4.9 to 14.2) compared with placebo to a similar degree as canagliflozin (Table 3). The SDs of log-transformed change in uEGF/Cr were 0.67 (placebo) and 0.47 (dapagliflozin). Each two-fold increase in uEGF/Cr over 1-year follow-up was also independently associated with a subsequent lower risk of the kidney outcomes corresponding to a HR of 0.81 (95% CI, 0.75 to 0.88; Figure 4). Consistent with CANVAS-CREDENCE, a stronger inverse association between uEGF/Cr change and kidney outcomes was observed at higher baseline UACR levels in DAPA-CKD, despite limited numbers in lower-UACR subgroups (*P* for interaction = 0.03). This association also differed by treatment allocation (*P* for interaction < 0.001), with a stronger association observed in participants treated with dapagliflozin (HR, 0.42; 95% CI, 0.30 to 0.60) compared with placebo (HR, 0.84; 95% CI, 0.75 to 0.93). In DAPA-CKD, analysis of annual eGFR slopes from baseline to month 36 showed a pattern consistent with CANVAS-CREDENCE, with participants in the lowest uEGF/Cr quartile experiencing a mean annual eGFR decline of −3.84 ml/min per 1.73 m^2^ (SEM, 0.20), compared with −2.59 ml/min per 1.73 m^2^ (SEM, 0.20) in the highest quartile (Figure 3). The distribution of uEGF/Cr at baseline was similar between patients with and without diabetes (Figure 5) The risk relationship between uEGF/Cr at baseline with kidney outcomes was also similar between participants with and without type 2 diabetes (Figure 5). Similarly, associations between changes in uEGF/Cr from baseline to year 1 and kidney outcomes were consistent regardless of diabetes status (Supplemental Figure 3).

**Figure 5:**
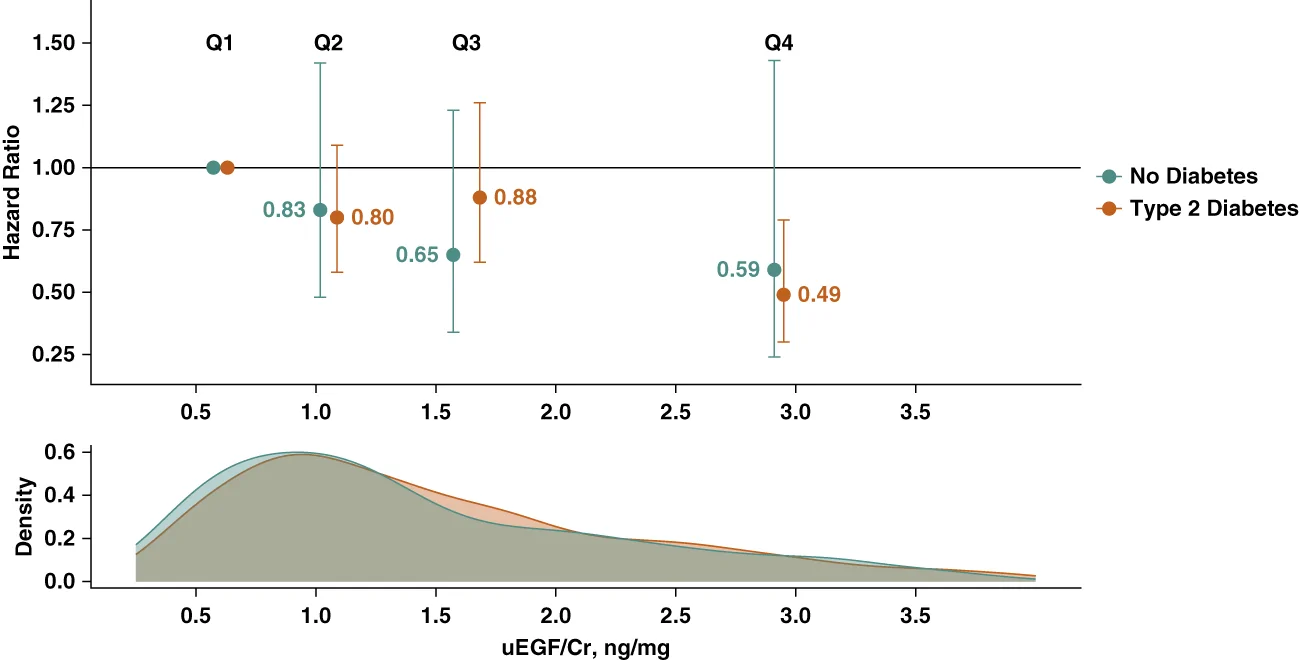
**Baseline association between uEGF/Cr quartiles and kidney outcomes in DAPA-CKD participants with and without type 2 diabetes, and the distribution of uEGF/Cr concentrations.** Association is shown between uEGF/Cr and the kidney outcome when uEGF/Cr is modeled as a categorical (quartiles) variable, stratified by diabetes status. Dots represent HRs for each quartile at the median concentration, bars indicate 95% CI. Model is adjusted for the following covariates: age, sex, race, randomized treatment, history of cardiovascular disease, HbA1c, smoking, systolic BP, BMI, eGFR, and log-transformed UACR.

## Discussion

In this *post hoc* analysis of participants from CANVAS and CREDENCE, and replicated in DAPA-CKD, we demonstrated that uEGF/Cr is consistently and independently associated with kidney outcomes and eGFR decline across a broad range of CKD severity. In addition, we showed that treatment with the SGLT2i canagliflozin and dapagliflozin attenuates the decline in uEGF/Cr over time. Changes in uEGF/Cr during the first year were independently associated with subsequent kidney outcomes, beyond changes in albuminuria, underscoring its potential utility for dynamic risk stratification.

Our findings confirm and extend previous observations of lower uEGF/Cr levels in patients with more advanced CKD, characterized by lower eGFR and higher albuminuria.^8^ Consistent with earlier studies, baseline uEGF/Cr was strongly inversely associated with kidney outcomes and annual eGFR slope decline. Adding uEGF/Cr to a comprehensive clinical model statistically significantly increased model fit and the C-statistic, but discrimination was already high and the absolute improvement was minimal and not clinically meaningful. Unlike previous analyses limited to more selective populations, our larger and more representative cohort included patients with a broad range of CKD and individuals with and without diabetes, allowing us to demonstrate that the association between uEGF/Cr and kidney outcomes is robust across all stages of eGFR and albuminuria.^17^ Consistent with previous observations, baseline uEGF/Cr decreased with higher UACR across eGFR categories, reflecting the link between impaired tubular health and glomerular damage. In addition, most previous studies measured uEGF in diabetes cohorts. We now also demonstrate that the associations between uEGF/Cr or changes in uEGF/Cr with kidney outcomes are consistent in patients with and without type 2 diabetes, noting that those without mostly had high albuminuria in our cohort. This supports the role of uEGF/Cr as a broadly applicable biomarker reflecting tubular regenerative capacity and kidney health.

Other translational biomarkers, including kidney injury molecule-1, tumor necrosis factor receptor (TNFR)-1, and TNFR-2, have demonstrated similar prognostic value for CKD progression. However, uEGF/Cr is distinct in reflecting tubular regenerative capacity rather than inflammation, and, unlike TNFR-1 and TNFR-2, shows early changes in response to SGLT2 inhibition, similar to kidney injury molecule-1.^6,18^

Previous studies have examined the role of EGF in the pathophysiology of CKD. These studies showed that lower uEGF/Cr levels likely indicate impaired distal tubular regenerative capacity and a shift toward defective repair processes that promote interstitial fibrosis and progressive kidney function loss.^3,19^ As a marker released predominantly from the distal tubule and thick ascending limb, uEGF reflects functional tubular mass and regenerative potential. It is therefore believed that declining levels signal exhaustion of kidney repair mechanisms.^6,20^ Conversely, preservation or increase of uEGF/Cr, as observed with SGLT2 inhibition, may reflect sustained tubular integrity and enhanced epithelial repair, consistent with the molecular reprogramming seen along all epithelial compartments of the kidney.^6,20–22^

Treatment with canagliflozin modestly attenuated the decline in uEGF/Cr over 1-year follow-up, consistently across baseline kidney function, albuminuria, and glycemic control. Given the modest magnitude of this effect, particularly relative to risk estimates expressed per two-fold increase, these findings are most informative for understanding SGLT2i effects on tubular health. Notably, this attenuation was sustained over 3 years in CREDENCE, suggesting a durable effect of SGLT2 inhibition on tubular health. These observations are in line with emerging evidence that SGLT2i exert nephroprotective effects, at least in part, by preserving tubular integrity.^6^ Interindividual variability in uEGF/Cr change was considerable, with approximately two-fold variation in the placebo arm, indicating that the observed treatment effect reflects a shift in distribution. The relative nephroprotective effect of canagliflozin on kidney outcomes was consistent across baseline uEGF/Cr tertiles, with numerically larger absolute risk reductions in participants with lower uEGF/Cr, reflecting higher baseline risk.

The association between changes in uEGF/Cr from baseline to year 1 adds a dynamic aspect to kidney risk stratification, consistent with the growing recognition of tubular markers as valuable prognostic tools in CKD.^9^ Increases in uEGF/Cr during treatment were independently associated with a significantly reduced risk of kidney outcomes. A novel finding of our study is the stronger association observed in participants with severely increased albuminuria, suggesting that patients with more advanced glomerular damage may derive greater prognostic benefit from monitoring tubular biomarker changes.

We replicated these findings in DAPA-CKD, where uEGF/Cr was similarly associated with kidney outcomes, and dapagliflozin treatment increased uEGF/Cr levels compared with placebo in a robust manner. Consistent with CANVAS-CREDENCE, changes in uEGF/Cr at 1 year were independently associated with kidney outcomes. Moreover, analysis of annual eGFR slope revealed a comparable pattern of less pronounced decline among participants with higher uEGF/Cr levels, supporting a robust association with better preserved kidney function. These findings demonstrate that uEGF/Cr is consistently associated with kidney outcomes and treatment effects across varied CKD populations, regardless of diabetes status or SGLT2i therapy, mostly reflecting high-albuminuria participants without diabetes. In CANVAS-CREDENCE, associations between changes in uEGF/Cr and kidney outcomes were consistent across treatment groups, whereas in DAPA-CKD we observed a significant interaction, with stronger associations among those receiving dapagliflozin, possibly reflecting greater potential for tubular protection in this higher-risk population.

Our findings contrast with those from the Study of Heart and Kidney Protection with Empagliflozin (EMPA-KIDNEY) trial, where the SGLT2i empagliflozin did not significantly change uEGF/Cr.^23^ These differences could potentially reflect variations in study design, baseline albuminuria, and diabetes status. Participants in EMPA-KIDNEY had lower baseline UACR compared with our DAPA-CKD cohort, where treatment effects on uEGF/Cr were more pronounced in participants with higher albuminuria. Consistently, in EMPA-KIDNEY, significant relative increases in uEGF/Cr with empagliflozin were observed in participants with UACR ≥1000 mg/g. Fewer participants had type 2 diabetes in EMPA-KIDNEY, and significant relative increases in uEGF/Cr were observed in those with diabetes only. Similarly, in DAPA-CKD, relative increases were also greatest among participants with type 2 diabetes.^24^

Our study has several limitations. As a *post hoc* observational analysis, causality cannot be inferred and residual confounding remains possible despite extensive covariate adjustment. The use of research-only assays precludes assuming calibration across lots, potentially contributing to observed interstudy differences. In addition, although we demonstrated consistent associations and treatment effects across three large trials, prospective validation in clinical practice is needed to confirm its utility for therapy guidance. Finally, while we included multiple diverse cohorts, heterogeneity in population characteristics may still limit generalizability. The practical value in clinical care and implementation is currently being investigated within the European Union–funded Personalized Drug Response: Implementation and Evaluation in CKD consortium.^25^

In summary, uEGF/Cr is a robust and consistent biomarker of tubular health, independently associated with kidney outcomes and eGFR decline across a wide CKD spectrum. Treatment with SGLT2i attenuates the decline in uEGF/Cr, and changes in this biomarker from baseline to year 1 provide substantial prognostic information, independent of albuminuria. Replication in DAPA-CKD underscores the potential of uEGF/Cr as a valuable tool for dynamic risk assessment and monitoring across diverse CKD populations and SGLT2i treatments.

## Supplementary Material

## Data Availability

Original data generated for the study will be made available upon reasonable request to the corresponding author. Data Type: Clinical Trial Data. Reason for Restricted Access: Data are subject to restrictions due to third-party ownership and data-sharing agreements with the sponsoring pharmaceutical companies.

## Supplemental Material

Supplemental Table 1. Baseline characteristics of placebo- and canagliflozin-treated participants from CANVAS and CREDENCE.

Supplemental Table 2. Baseline characteristics of placebo- and dapagliflozin-treated participants from DAPA-CKD.

Supplemental Table 3. Effect of canagliflozin compared with placebo on uEGF/Cr over time in participants from the CREDENCE trial.

Supplemental Table 4. Relative and absolute risk reductions for the kidney outcome with SGLT2i treatment compared with placebo across baseline uEGF/Cr tertiles.

Supplemental Figure 1. Association between uEGF/Cr and kidney outcome at baseline by eGFR and UACR subgroups in CANVAS-CREDENCE.

Supplemental Figure 2. Association of change in uEGF/Cr from baseline to year 1 with kidney outcome by eGFR and UACR subgroups in CANVAS-CREDENCE.

Supplemental Figure 3. Associations of change in uEGF/Cr from baseline to year 1 with kidney outcomes in DAPA-CKD participants with and without type 2 diabetes, and the distribution of uEGF/Cr changes.
