# Supplementary material for: Association of Urinary EGF with Kidney Outcomes and Effects of Sodium-Glucose Cotransporter 2 Inhibition
Source: J Am Soc Nephrol. 2026 Apr 2;37(10):2210–20. doi: 10.1681/ASN.0000001086 (PMC13641454; doi:10.1681/ASN.0000001086)
Supplement: Supplementary file 1 [file jasn-37-2210-s001.pdf]

ASN American Society of Nephrology
JASN Journal of the American Society of Nephrology
KIDNEY360 Accessing Our World From Every Angle
CJASN Clinical Journal of the American Society of Nephrology

ASN Journal Disclosure Form

As per ASN journal policy, I have disclosed any financial relationships or commitments I have held in the past 36 months as included below. I have listed my Current Employer below to indicate there is a relationship requiring disclosure. If no relationship exists, my Current Employer is not listed.

C. Arnott reports the following:
Employer: The George Institute for Global Health; NSW Health/ Sydney Local Health District; Research Funding: Abbott Vascular; Honoraria: Amgen; Astra Zeneca, Novo Nordisk; and Advisory or Leadership Role: Novo Nordisk; Astra Zeneca.

I understand that the information above will be published within the journal article, if accepted, and that failure to comply and/or to accurately and completely report the potential financial conflicts of interest could lead to the following: 1) Prior to publication, article rejection, or 2) Post-publication, sanctions ranging from, but not limited to, issuing a correction, reporting the inaccurate information to the authors' institution, banning authors from submitting work to ASN journals for varying lengths of time, and/or retraction of the published work.

Name: Clare Gabrielle Arnott
Manuscript ID: JASN-2025-001540R1
Manuscript Title: Association of Urinary Epidermal Growth Factor with Kidney Outcomes and Effects of SGLT2 Inhibition,
Date of Completion: February 17, 2026
Disclosure Updated Date: June 18, 2025

ASN
American Society of Nephrology
JASN
Journal of the American Society of Nephrology
KIDNEY360
Accessing Our World From Every Angle
CJASN
Clinical Journal of the American Society of Nephrology

ASN Journal Disclosure Form

As per ASN journal policy, I have disclosed any financial relationships or commitments I have held in the past 36 months as included below. I have listed my Current Employer below to indicate there is a relationship requiring disclosure. If no relationship exists, my Current Employer is not listed.

S. Bakker reports the following:
Employer: University Medical Center Groningen; and Research Funding: Astellas Pharma, Chiesi, bioMerieux.

I understand that the information above will be published within the journal article, if accepted, and that failure to comply and/or to accurately and completely report the potential financial conflicts of interest could lead to the following: 1) Prior to publication, article rejection, or 2) Post-publication, sanctions ranging from, but not limited to, issuing a correction, reporting the inaccurate information to the authors' institution, banning authors from submitting work to ASN journals for varying lengths of time, and/or retraction of the published work.

Name: Stephan J.L. Bakker
Manuscript ID: JASN-2025-001540R1
Manuscript Title: Association of Urinary Epidermal Growth Factor with Kidney Outcomes and Effects of SGLT2 Inhibition
Date of Completion: February 17, 2026
Disclosure Updated Date: May 9, 2025

ASN American Society of Nephrology JASN Journal of the American Society of Nephrology KIDNEY360 Accessing Our World From Every Angle CJASN Clinical Journal of the American Society of Nephrology

ASN Journal Disclosure Form

As per ASN journal policy, I have disclosed any financial relationships or commitments I have held in the past 36 months as included below. I have listed my Current Employer below to indicate there is a relationship requiring disclosure. If no relationship exists, my Current Employer is not listed.

P. Bjornstad reports the following:
Employer: University of Washington; Consultancy: AstraZeneca; Boehringer Ingelheim; Bayer; Horizon Pharma; XORTX; Novo Nordisk; Lilly; Roche; Sidera Bio; Research Funding: Horizon Pharma/Amgen, AstraZeneca, Merck, Novo Nordisk, Lilly; and Advisory or Leadership Role: Horizon Pharma/Amgen, XORTX, AstraZeneca, Boehringer-Ingelheim, Bayer, Lilly, Novo Nordisk; Roche.

I understand that the information above will be published within the journal article, if accepted, and that failure to comply and/or to accurately and completely report the potential financial conflicts of interest could lead to the following: 1) Prior to publication, article rejection, or 2) Post-publication, sanctions ranging from, but not limited to, issuing a correction, reporting the inaccurate information to the authors' institution, banning authors from submitting work to ASN journals for varying lengths of time, and/or retraction of the published work.

Name: Petter Bjornstad
Manuscript ID: JASN-2025-001540R1
Manuscript Title: Association of Urinary Epidermal Growth Factor with Kidney Outcomes and Effects of SGLT2 Inhibition
Date of Completion: February 22, 2026
Disclosure Updated Date: February 22, 2026

ASN American Society of Nephrology
JASN Journal of the American Society of Nephrology
KIDNEY360 Accessing Our World From Every Angle
CJASN Clinical Journal of the American Society of Nephrology

ASN Journal Disclosure Form

As per ASN journal policy, I have disclosed any financial relationships or commitments I have held in the past 36 months as included below. I have listed my Current Employer below to indicate there is a relationship requiring disclosure. If no relationship exists, my Current Employer is not listed.

S. Coca reports the following:
Employer: Icahn School of Medicine at Mount Sinai; Mount Sinai owns part of Renalytix; Consultancy: Renalytix, Bayer, Alexion, SC Pharma, Whiteswell, Vera Therapeutics, Nuwellis; Ownership Interest: Renalytix; Research Funding: Renalytix, ProKidney; Patents or Royalties: Renalytix; and Other Interests or Relationships: Associate Editor for Kidney360, Editorial Boards of JASN, CJASN, Kidney International.

I understand that the information above will be published within the journal article, if accepted, and that failure to comply and/or to accurately and completely report the potential financial conflicts of interest could lead to the following: 1) Prior to publication, article rejection, or 2) Post-publication, sanctions ranging from, but not limited to, issuing a correction, reporting the inaccurate information to the authors' institution, banning authors from submitting work to ASN journals for varying lengths of time, and/or retraction of the published work.

Name: Steven G. Coca
Manuscript ID: JASN-2025-001540R1
Manuscript Title: Association of Urinary Epidermal Growth Factor with Kidney Outcomes and Effects of SGLT2 Inhibition
Date of Completion: February 17, 2026
Disclosure Updated Date: October 3, 2025

ASN Journal Disclosure Form

As per ASN journal policy, I have disclosed any financial relationships or commitments I have held in the past 36 months as included below. I have listed my Current Employer below to indicate there is a relationship requiring disclosure. If no relationship exists, my Current Employer is not listed.

P. Greasley reports the following:
Employer: AstraZeneca; Ownership Interest: AstraZeneca; and Patents or Royalties: AstraZeneca.

I understand that the information above will be published within the journal article, if accepted, and that failure to comply and/or to accurately and completely report the potential financial conflicts of interest could lead to the following: 1) Prior to publication, article rejection, or 2) Post-publication, sanctions ranging from, but not limited to, issuing a correction, reporting the inaccurate information to the authors' institution, banning authors from submitting work to ASN journals for varying lengths of time, and/or retraction of the published work.

Name: Peter J. Greasley
Manuscript ID: JASN-2025-001540R1
Manuscript Title: Association of Urinary Epidermal Growth Factor with Kidney Outcomes and Effects of SGLT2 Inhibition
Date of Completion: February 25, 2026
Disclosure Updated Date: February 25, 2026

ASN American Society of Nephrology
JASN Journal of the American Society of Nephrology
KIDNEY360 Accessing Our World From Every Angle
CJASN Clinical Journal of the American Society of Nephrology

ASN Journal Disclosure Form

As per ASN journal policy, I have disclosed any financial relationships or commitments I have held in the past 36 months as included below. I have listed my Current Employer below to indicate there is a relationship requiring disclosure. If no relationship exists, my Current Employer is not listed.

A. Hammarstedt reports the following:
Employer: AstraZeneca; and Ownership Interest: AstraZeneca.

I understand that the information above will be published within the journal article, if accepted, and that failure to comply and/or to accurately and completely report the potential financial conflicts of interest could lead to the following: 1) Prior to publication, article rejection, or 2) Post-publication, sanctions ranging from, but not limited to, issuing a correction, reporting the inaccurate information to the authors' institution, banning authors from submitting work to ASN journals for varying lengths of time, and/or retraction of the published work.

Name: Ann Hammarstedt
Manuscript ID: JASN-2025-001540R3
Manuscript Title: Association of Urinary Epidermal Growth Factor with Kidney Outcomes and Effects of SGLT2 Inhibition
Date of Completion: March 20, 2026
Disclosure Updated Date: February 18, 2026

ASN American Society of Nephrology | JASN Journal of the American Society of Nephrology | KIDNEY360 Accessing Our World From Every Angle | CJASN Clinical Journal of the American Society of Nephrology

ASN Journal Disclosure Form

As per ASN journal policy, I have disclosed any financial relationships or commitments I have held in the past 36 months as included below. I have listed my Current Employer below to indicate there is a relationship requiring disclosure. If no relationship exists, my Current Employer is not listed.

M. Hansen reports the following:
Employer: Janssen Research & Development; and Ownership Interest: Johnson & Johnson.

I understand that the information above will be published within the journal article, if accepted, and that failure to comply and/or to accurately and completely report the potential financial conflicts of interest could lead to the following: 1) Prior to publication, article rejection, or 2) Post-publication, sanctions ranging from, but not limited to, issuing a correction, reporting the inaccurate information to the authors' institution, banning authors from submitting work to ASN journals for varying lengths of time, and/or retraction of the published work.

Name: Michael K. Hansen
Manuscript ID: JASN-2025-001540R1
Manuscript Title: Association of Urinary Epidermal Growth Factor with Kidney Outcomes and Effects of SGLT2 Inhibition
Date of Completion: February 17, 2026
Disclosure Updated Date: February 17, 2026

ASN American Society of Nephrology | JASN Journal of the American Society of Nephrology | KIDNEY360 Accessing Our World From Every Angle | CJASN Clinical Journal of the American Society of Nephrology

ASN Journal Disclosure Form

As per ASN journal policy, I have disclosed any financial relationships or commitments I have held in the past 36 months as included below. I have listed my Current Employer below to indicate there is a relationship requiring disclosure. If no relationship exists, my Current Employer is not listed.

H. Heerspink reports the following:
Employer: University Medical Center Groningen; Consultancy: Ongoing consultancy agreements with AstraZeneca, Alexion, Alnylum, Bayer, Boehringer Ingelheim, Biocity Biopharmaceuticals, Dimerix, Eli-Lilly, Novartis, NovoNordisk, Otsuka, Roche, Travere Therapeutics; Research Funding: AstraZeneca, Bayer, Boehringer Ingelheim, and NovoNordisk research support (grant funding directed to employer); Honoraria: Lecture fees from AstraZeneca, Bayer, Eli-Lilly and NovoNordisk; and Speakers Bureau: AstraZeneca, Bayer, Novo Nordisk.

I understand that the information above will be published within the journal article, if accepted, and that failure to comply and/or to accurately and completely report the potential financial conflicts of interest could lead to the following: 1) Prior to publication, article rejection, or 2) Post-publication, sanctions ranging from, but not limited to, issuing a correction, reporting the inaccurate information to the authors' institution, banning authors from submitting work to ASN journals for varying lengths of time, and/or retraction of the published work.

Name: Hiddo Jan L. Heerspink
Manuscript ID: JASN-2025-001540R1
Manuscript Title: Association of Urinary Epidermal Growth Factor with Kidney Outcomes and Effects of SGLT2 Inhibition
Date of Completion: February 17, 2026
Disclosure Updated Date: February 7, 2026

ASN Journal Disclosure Form

As per ASN journal policy, I have disclosed any financial relationships or commitments I have held in the past 36 months as included below. I have listed my Current Employer below to indicate there is a relationship requiring disclosure. If no relationship exists, my Current Employer is not listed.

N. Jongs reports the following:
Employer: UMCG; and Speakers Bureau: AstraZeneca.

I understand that the information above will be published within the journal article, if accepted, and that failure to comply and/or to accurately and completely report the potential financial conflicts of interest could lead to the following: 1) Prior to publication, article rejection, or 2) Post-publication, sanctions ranging from, but not limited to, issuing a correction, reporting the inaccurate information to the authors' institution, banning authors from submitting work to ASN journals for varying lengths of time, and/or retraction of the published work.

Name: Niels Jongs
Manuscript ID: JASN-2025-001540R3
Manuscript Title: Association of Urinary Epidermal Growth Factor with Kidney Outcomes and Effects of SGLT2 Inhibition
Date of Completion: March 20, 2026
Disclosure Updated Date: May 22, 2025

ASN
American Society of Nephrology
JASN
Journal of the American Society of Nephrology
KIDNEY360
Accessing Our World From Every Angle
CJASN
Clinical Journal of the American Society of Nephrology

ASN Journal Disclosure Form

As per ASN journal policy, I have disclosed any financial relationships or commitments I have held in the past 36 months as included below. I have listed my Current Employer below to indicate there is a relationship requiring disclosure. If no relationship exists, my Current Employer is not listed.

W. Ju reports the following:
Employer: University of Michigan (Relationship has not ended); Consultancy: TRAVERE (Relationship has not ended); Research Funding: Eli Lilly (Relationship has not ended); Novo Nordisk (Relationship has not ended); Sanofi (Relationship has not ended); Roche (Relationship has not ended); Boehringer Ingelheim (Relationship has not ended); Bayer (Relationship has not ended); and Patents or Royalties: AstraZeneca (Relationship has not ended).

I understand that the information above will be published within the journal article, if accepted, and that failure to comply and/or to accurately and completely report the potential financial conflicts of interest could lead to the following: 1) Prior to publication, article rejection, or 2) Post-publication, sanctions ranging from, but not limited to, issuing a correction, reporting the inaccurate information to the authors' institution, banning authors from submitting work to ASN journals for varying lengths of time, and/or retraction of the published work.

Name: Wenjun Ju
Manuscript ID: #JASN-2025-001540R3
Manuscript Title: Association of Urinary Epidermal Growth Factor with Kidney Outcomes and Effects of SGLT2 Inhibition
Date of Completion: March 18, 2026
Disclosure Updated Date: March 17, 2026

American Society of Nephrology
JASN Journal of the American Society of Nephrology
KIDNEY360 Accessing Our World From Every Angle
CJASN Clinical Journal of the American Society of Nephrology

ASN Journal Disclosure Form

As per ASN journal policy, I have disclosed any financial relationships or commitments I have held in the past 36 months as included below. I have listed my Current Employer below to indicate there is a relationship requiring disclosure. If no relationship exists, my Current Employer is not listed.

A. Koshino reports the following:
Employer: Kanazawa University; and Speakers Bureau: Astrazeneca, Kyowa Kirin, Terumo.

I understand that the information above will be published within the journal article, if accepted, and that failure to comply and/or to accurately and completely report the potential financial conflicts of interest could lead to the following: 1) Prior to publication, article rejection, or 2) Post-publication, sanctions ranging from, but not limited to, issuing a correction, reporting the inaccurate information to the authors' institution, banning authors from submitting work to ASN journals for varying lengths of time, and/or retraction of the published work.

Name: Akihiko Koshino
Manuscript ID: JASN-2025-001540R1
Manuscript Title: Association of Urinary Epidermal Growth Factor with Kidney Outcomes and Effects of SGLT2 Inhibition,
Date of Completion: February 17, 2026
Disclosure Updated Date: February 17, 2026

ASN American Society of Nephrology | JASN Journal of the American Society of Nephrology | KIDNEY360 Accessing Our World From Every Angle | CJASN Clinical Journal of the American Society of Nephrology

ASN Journal Disclosure Form

As per ASN journal policy, I have disclosed any financial relationships or commitments I have held in the past 36 months as included below. I have listed my Current Employer below to indicate there is a relationship requiring disclosure. If no relationship exists, my Current Employer is not listed.

M. Kretzler reports the following:
Employer: University of Michigan Medical School; Consultancy: None by me personally.; As employee of U Michigan for: Janssen, NovoNordisk, Otsuka, Alexion/Astra-Zeneca, Variant Bio, Novartis, Vera.; Research Funding: Sponsored research project as PI at U Michigan: National Institutes of Health, Chan Zuckerberg Initiative, Breakthrough T1D, Alport Foundation, AstraZeneca, NovoNordisk, Eli Lilly, Bayer, Boehringer-Ingelheim, European Union Innovative Medicine Initiative, Certa, RenalytixAI, Travere, Regeneron, Maze Therapeutics, Roche-Genentech, Sanofi, Dimerix, Vera Therapeutics.; Patents or Royalties: PCT/EP2014/073413: Biomarkers and methods for progression prediction for chronic kidney disease.; Royalty sharing Astra-Zeneca CKD drug development and drug development revenue from Astra-Zeneca via U Michigan.; and Advisory or Leadership Role: Editorial Board: J Am Soc Nephrology, Kidney International, Kidney Disease; Advisory Board Chair: Nephcure.

I understand that the information above will be published within the journal article, if accepted, and that failure to comply and/or to accurately and completely report the potential financial conflicts of interest could lead to the following: 1) Prior to publication, article rejection, or 2) Post-publication, sanctions ranging from, but not limited to, issuing a correction, reporting the inaccurate information to the authors' institution, banning authors from submitting work to ASN journals for varying lengths of time, and/or retraction of the published work.

Name: Matthias Kretzler
Manuscript ID: JASN-2025-001540R1
Manuscript Title: Association of Urinary Epidermal Growth Factor with Kidney Outcomes and Effects of SGLT2 Inhibition.
Date of Completion: March 2, 2026
Disclosure Updated Date: March 2, 2026

ASN American Society of Nephrology | JASN Journal of the American Society of Nephrology | KIDNEY360 Accessing Our World From Every Angle | CJASN Clinical Journal of the American Society of Nephrology

ASN Journal Disclosure Form

As per ASN journal policy, I have disclosed any financial relationships or commitments I have held in the past 36 months as included below. I have listed my Current Employer below to indicate there is a relationship requiring disclosure. If no relationship exists, my Current Employer is not listed.

E. Moedt reports the following:
Employer: University Medical Center Groningen

I understand that the information above will be published within the journal article, if accepted, and that failure to comply and/or to accurately and completely report the potential financial conflicts of interest could lead to the following: 1) Prior to publication, article rejection, or 2) Post-publication, sanctions ranging from, but not limited to, issuing a correction, reporting the inaccurate information to the authors' institution, banning authors from submitting work to ASN journals for varying lengths of time, and/or retraction of the published work.

Name: Erik Moedt
Manuscript ID: JASN-2025-001540R1
Manuscript Title: Association of Urinary Epidermal Growth Factor with Kidney Outcomes and Effects of SGLT2 Inhibition
Date of Completion: February 16, 2026
Disclosure Updated Date: February 16, 2026

ASN
American Society of Nephrology
JASN
Journal of the American Society of Nephrology
KIDNEY360
Accessing Our World From Every Angle
CJASN
Clinical Journal of the American Society of Nephrology

ASN Journal Disclosure Form

As per ASN journal policy, I have disclosed any financial relationships or commitments I have held in the past 36 months as included below. I have listed my Current Employer below to indicate there is a relationship requiring disclosure. If no relationship exists, my Current Employer is not listed.

V. Nair reports the following:
Employer: University of Michigan

I understand that the information above will be published within the journal article, if accepted, and that failure to comply and/or to accurately and completely report the potential financial conflicts of interest could lead to the following: 1) Prior to publication, article rejection, or 2) Post-publication, sanctions ranging from, but not limited to, issuing a correction, reporting the inaccurate information to the authors' institution, banning authors from submitting work to ASN journals for varying lengths of time, and/or retraction of the published work.

Name: Viji Nair
Manuscript ID: JASN-2025-001540R1
Manuscript Title: Association of Urinary Epidermal Growth Factor with Kidney Outcomes and Effects of SGLT2 Inhibition
Date of Completion: February 24, 2026
Disclosure Updated Date: May 2, 2025

ASN American Society of Nephrology | JASN Journal of the American Society of Nephrology | KIDNEY360 Accessing Our World From Every Angle | CJASN Clinical Journal of the American Society of Nephrology

ASN Journal Disclosure Form

As per ASN journal policy, I have disclosed any financial relationships or commitments I have held in the past 36 months as included below. I have listed my Current Employer below to indicate there is a relationship requiring disclosure. If no relationship exists, my Current Employer is not listed.

P. Rossing reports the following:
Employer: Steno Diabetes Center Copenhagen; Research Funding: Novo Nordisk , AstraZeneca, Bayer, Lexicon Pharma; Honoraria: Boehringer Ingelheim, AstraZeneca, Abbott, Novo Nordisk, all honoraria to institution; and Advisory or Leadership Role: Astra Zeneca Bayer , Novo Nordisk, Gilead all honoraria to institution.

I understand that the information above will be published within the journal article, if accepted, and that failure to comply and/or to accurately and completely report the potential financial conflicts of interest could lead to the following: 1) Prior to publication, article rejection, or 2) Post-publication, sanctions ranging from, but not limited to, issuing a correction, reporting the inaccurate information to the authors' institution, banning authors from submitting work to ASN journals for varying lengths of time, and/or retraction of the published work.

Name: Peter Rossing
Manuscript ID: JASN-2025-001540R1
Manuscript Title: Association of Urinary Epidermal Growth Factor with Kidney Outcomes and Effects of SGLT2 Inhibition
Date of Completion: February 17, 2026
Disclosure Updated Date: March 19, 2025

ASN American Society of Nephrology
JASN Journal of the American Society of Nephrology
KIDNEY360 Accessing Our World From Every Angle
CJASN Clinical Journal of the American Society of Nephrology

ASN Journal Disclosure Form

As per ASN journal policy, I have disclosed any financial relationships or commitments I have held in the past 36 months as included below. I have listed my Current Employer below to indicate there is a relationship requiring disclosure. If no relationship exists, my Current Employer is not listed.

D. Wheeler reports the following:
Employer: University College London; Consultancy: Fees from: Astellas, AstraZeneca, Bayer, Boehringer Ingelheim, Dimerix, Eledon, Galderma, George Clinical, Gilead, GlaxoSmithKline, Janssen, Merck Sharp and Dohme, Mineralys, Pathalys, Pfizer, ProKidney, Sana, Silence, Synthekine, Tricida, Vifor, Vertex, Zydus for Talks, Advisory Boards, Trial Committees and Consultancy; Research Funding: National Institute for Health Research; Kidney Research UK; Honoraria: Astellas, AstraZeneca, Bayer, Boehringer Ingelheim, Eledon, Galderma, George Clinical, GlaxoSmithKline, Merck Sharp and Dohme, Mineralys, Pathalys, ProKidney, Sana, Silence, Synthekine, Vifor, Vertex,; Advisory or Leadership Role: National Institute of Health Research Clinical Lead for Renal Disorders.; International society of Nephrology; American Journal of Kidney Disease; and Speakers Bureau: AstraZeneca, Astellas.

I understand that the information above will be published within the journal article, if accepted, and that failure to comply and/or to accurately and completely report the potential financial conflicts of interest could lead to the following: 1) Prior to publication, article rejection, or 2) Post-publication, sanctions ranging from, but not limited to, issuing a correction, reporting the inaccurate information to the authors' institution, banning authors from submitting work to ASN journals for varying lengths of time, and/or retraction of the published work.

Name: David C. Wheeler
Manuscript ID: #JASN-2025-001540R1
Manuscript Title: Association of Urinary Epidermal Growth Factor with Kidney Outcomes and Effects of SGLT2 Inhibition
Date of Completion: February 16, 2026
Disclosure Updated Date: May 28, 2025
