# Supplementary material for: Association of Urinary EGF with Kidney Outcomes and Effects of Sodium-Glucose Cotransporter 2 Inhibition
Source: J Am Soc Nephrol. 2026 Apr 2;37(10):2210–20. doi: 10.1681/ASN.0000001086 (PMC13641454; doi:10.1681/ASN.0000001086)
Supplement: Supplementary file 2 [file jasn-37-2210-s002.pdf]

**Supplemental Material**

**Supplemental Table 1:** Baseline characteristics of placebo- and canagliflozin-treated participants from CANVAS and CREDENCE........ 2

**Supplemental Table 2:** Baseline characteristics of placebo- and dapagliflozin-treated participants from DAPA-CKD. 2

**Supplemental Table 3:** Effect of canagliflozin compared to placebo on uEGF/Cr over time in participants from the CREDENCE trial........4

**Supplemental Table 4:** Relative and absolute risk reductions for the kidney outcome with SGLT2i treatment (canagliflozin 100 mg or dapagliflozin 10 mg) compared to placebo across baseline uEGF/Cr tertiles........5

**Supplemental Figure 1:** Association between uEGF/Cr and kidney outcome at baseline by eGFR and UACR subgroups in CANVAS-CREDENCE........ 6

**Supplemental Figure 2:** Association of change in uEGF/Cr from baseline to year 1 with kidney outcome by eGFR and UACR subgroups in CANVAS-CREDENCE........7

**Supplemental Figure 3:** Associations of change in uEGF/Cr from baseline to year 1 with kidney outcomes in DAPA-CKD participants with and without type 2 diabetes, and the distribution of uEGF/Cr changes. ........ 8

**Supplemental Table 1:** Baseline characteristics of placebo- and canagliflozin-treated participants from CANVAS and CREDENCE

| | CANVAS | | CREDENCE | |
|---|---|---|---|---|
| Characteristic | Canagliflozin (N=2340) | Placebo (N=1181) | Canagliflozin (N=1249) | Placebo (N=1208) |
| Age, years | 63 (7.8) | 63 (7.8) | 63 (9.1) | 63 (9.1) |
| Male sex, n (%) | 1562 (67%) | 790 (67%) | 829 (67%) | 805 (67%) |
| Race | | | | |
| Asian, n (%) | 231 (10%) | 120 (10%) | 159 (13%) | 166 (14%) |
| Black, n (%) | 50 (2%) | 30 (3%) | 63 (5%) | 52 (4%) |
| Other, n (%) | 154 (7%) | 73 (6%) | 123 (10%) | 127 (11%) |
| White, n (%) | 1905 (81%) | 958 (81%) | 904 (72%) | 863 (71%) |
| Smoking, n (%) | 400 (17%) | 239 (20%) | 184 (15%) | 184 (15%) |
| Diabetes duration, years | 13.7 (7.5) | 13.3 (7.5) | 15.8 (8.7) | 16.0 (8.4) |
| CVD history, n (%) | 1389 (59%) | 698 (59%) | 652 (52%) | 623 (52%) |
| BMI, kg/m$^2$ | 32.7 (6.11) | 32.6 (6.18) | 31.8 (6.18) | 31.8 (6.16) |
| Systolic BP, mmHg | 136 (15.9) | 137 (15.8) | 140 (15.8) | 140 (15.7) |
| $HbA_{1C}$, % | 8.18 (0.92) | 8.16 (0.91) | 8.27 (1.32) | 8.21 (1.28) |
| eGFR, ml/min/1.73 m$^2$ | 77.0 (18.7) | 76.9 (18.8) | 56.9 (18.2) | 57.0 (18.5) |
| UACR, mg/g (IQR) | 11.7 [6.50;34.4] | 11.5 [6.25;37.0] | 926 [464;1763] | 899 [460;1742] |
| uEGF, ng/ml (IQR) | 4.57 [2.59;7.75] | 4.56 [2.63;7.85] | 1.43 [0.82;2.52] | 1.47 [0.86;2.63] |
| uEGF/Cr, ng/mg (IQR) | 5.08 [3.32;7.91] | 5.22 [3.44;7.67] | 2.16 [1.36;3.50] | 2.20 [1.37;3.39] |

*Numerical variables with a normal distribution are presented as mean (SD). Variables with a skewed distribution, UACR, uEGF, and uEGF/Cr, are reported as median (IQR). Categorical variables are presented as frequency (%). Abbreviations: CVD: cardiovascular disease; BMI: body mass index; BP: blood pressure; HbA1c: hemoglobin $A_{1c}$;*
*eGFR: estimated glomerular filtration rate; UACR: urine albumin-creatinine ratio; IQR: interquartile range; uEGF: urinary epidermal growth factor; uEGF/Cr: urinary epidermal growth factor-creatinine ratio; SD: standard deviation.*

**Supplemental Table 2:** Baseline characteristics of placebo- and dapagliflozin-treated participants from DAPA-CKD

| Characteristic | Dapagliflozin (N=1246) | Placebo (N=1204) |
|---|---|---|
| Age, years | 62 (12.0) | 62 (12.1) |
| Male sex, n (%) | 833 (67%) | 803 (67%) |
| Race | | |
| Asian, n (%) | 285 (23%) | 251 (21%) |
| Black, n (%) | 50 (4%) | 42 (3%) |
| Other, n (%) | 144 (12%) | 141 (12%) |
| White, n (%) | 767 (62%) | 770 (64%) |
| Smoking, n (%) | 162 (13%) | 170 (14%) |
| Diabetes, n (%) | 864 (69%) | 829 (69%) |
| CVD history, n (%) | 494 (40%) | 479 (40%) |
| BMI, $kg/m^2$ | 30.1 (6.12) | 30.5 (6.44) |
| Systolic BP, mmHg | 137 (17.2) | 139 (17.6) |
| $HbA_{1C}$, % | 7.17 (1.77) | 7.09 (1.67) |
| eGFR, ml/min/1.73 $m^2$ | 43.7 (12.6) | 43.5 (12.5) |
| UACR, mg/g (IQR) | 937 [443;1870] | 946 [477;1901] |
| uEGF, ng/ml (IQR) | 0.98 [0.55;1.76] | 0.99 [0.52;1.77] |
| uEGF/Cr, ng/mg (IQR) | 1.32 [0.84;2.11] | 1.31 [0.86;2.17] |

*Numerical variables with a normal distribution are presented as mean (SD). Variables with a skewed distribution, UACR, uEGF, and uEGF/Cr, are reported as median (IQR). Categorical variables are presented as frequency (%). Abbreviations: CVD: cardiovascular disease; BMI: body mass index; BP: blood pressure; HbA1c: hemoglobin $A_{1c}$; eGFR: estimated glomerular filtration rate; UACR: urine albumin-creatinine ratio; IQR: interquartile range; uEGF: urinary epidermal growth factor; uEGF/Cr: urinary epidermal growth factor-creatinine ratio; SD: standard deviation.*

**Supplemental Table 3:** Effect of canagliflozin compared to placebo on uEGF/Cr over time in participants from the CREDENCE trial

| | Geometric mean baseline uEGF/Cr, ng/mg | Change from baseline to year 1, % (95% CI) | Change from baseline to year 3, % (95% CI) | Change over time, % (95% CI) | *P*-value* |
|---|---|---|---|---|---|
| Canagliflozin | 2.15 | **-5.4** (-8.7, -1.9) | **-14.4** (-20.1, -8.4) | **-7.6** (-10.8, -4.4) | <0.001 |
| Placebo | 2.14 | **-12.5** (-15.6, -9.1) | **-26.6** (-31.8, -20.9) | **-15.8** (-19.3, -13.2) | <0.001 |
| Between-group difference | | **8.1** (1.1, 15.6) | **16.5** (2.1, 32.8) | **9.8** (4.4, 15.4) | <0.001 |

*Treatment effect was estimated using a repeated measures model, adjusted for uEGF/Cr at baseline.*
*Abbreviations: uEGF/Cr: urinary epidermal growth factor-creatinine ratio; CI: confidence interval;*
**P value indicates the change over time*

**Supplemental Table 4:** Relative and absolute risk reductions for the kidney outcome with SGLT2i treatment (canagliflozin 100 mg or dapagliflozin 10 mg) compared to placebo across baseline uEGF/Cr tertiles

| | Events / Patients (%) | | Event rate (per 100 patient-years) | | | | |
|---|---|---|---|---|---|---|---|
| **Subgroup** | **SGLT2i** | **Placebo** | **SGLT2i** | **Placebo** | **HR (95% CI)** | **P for interaction** | **Absolute risk reduction, %** |
| **CANVAS-CREDENCE** | 147/2418 (6.1%) | 242/2389 (10.1%) | 0.34 | 0.64 | 0.55 (0.45 to 0.67) | 0.78 | 0.30 |
| Tertile 1 | 92/827 (11.1%) | 144/776 (18.6%) | 0.32 | 0.67 | 0.52 (0.40 to 0.68) | | 0.35 |
| Tertile 2 | 38/781 (4.9%) | 64/821 (7.8%) | 0.44 | 0.70 | 0.63 (0.42 to 0.94) | | 0.26 |
| Tertile 3 | 17/810 (2.1%) | 34/792 (4.3%) | 0.33 | 0.63 | 0.51 (0.28 to 0.93) | | 0.30 |
| **DAPA-CKD** | 155/1246 (12.4%) | 201/1204 (16.7%) | 5.89 | 8.08 | 0.67 (0.54 to 0.83) | 0.53 | 2.19 |
| Tertile 1 | 75/422 (17.8%) | 105/395 (26.6%) | 8.88 | 13.63 | 0.63 (0.46 to 0.85) | | 4.75 |
| Tertile 2 | 56/410 (13.7%) | 57/406 (14.0%) | 6.41 | 6.77 | 0.79 (0.55 to 1.16) | | 0.36 |
| Tertile 3 | 24/414 (11.2%) | 39/403 (9.7%) | 2.63 | 4.46 | 0.57 (0.34 to 0.97) | | 1.83 |

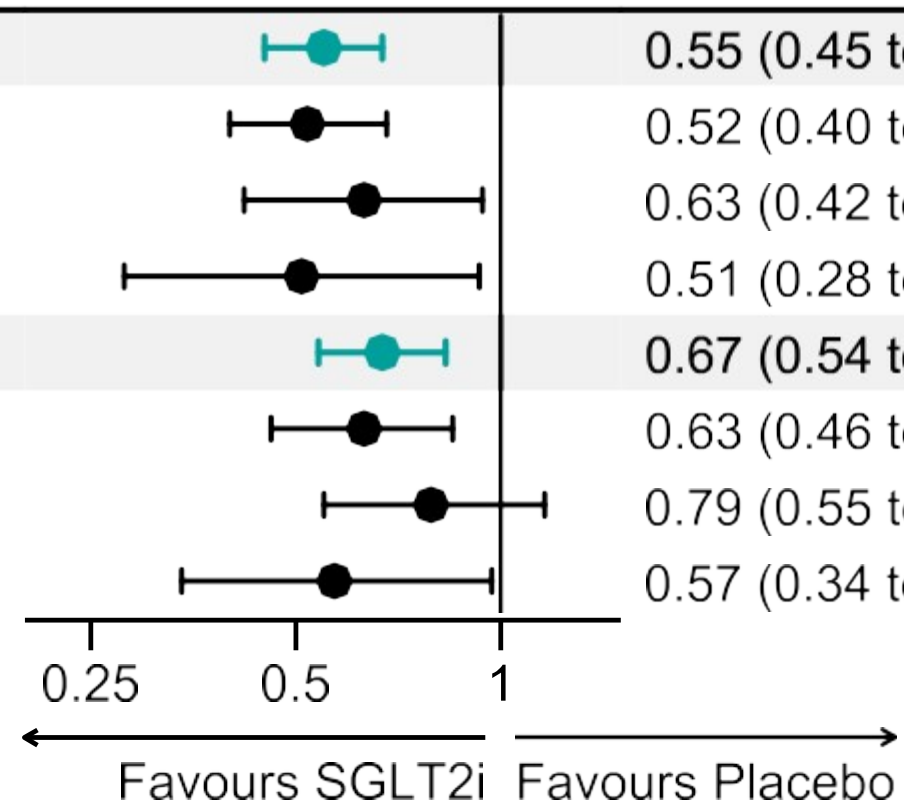


*Association is shown of treatment assignment with the kidney outcome by baseline uEGF/Cr tertiles.*

*HRs compare SGLT2i treatment versus placebo and are adjusted for age, sex, race, trial (not for DAPA-CKD), history of CVD, HbA1c, smoking, systolic BP, BMI, eGFR, and log-transformed UACR.*

*Abbreviations: CI: confidence interval; HR: hazard ratio; uEGF/Cr: urinary epidermal growth factor-creatinine ratio; CVD: cardiovascular disease; HbA1c: hemoglobin $A_{1c}$; BP: blood pressure; BMI: body mass index; eGFR: estimated glomerular filtration rate; UACR: urine albumin-creatinine ratio;*

**Supplemental Figure 1:** Association between uEGF/Cr and kidney outcome at baseline by eGFR and UACR subgroups in

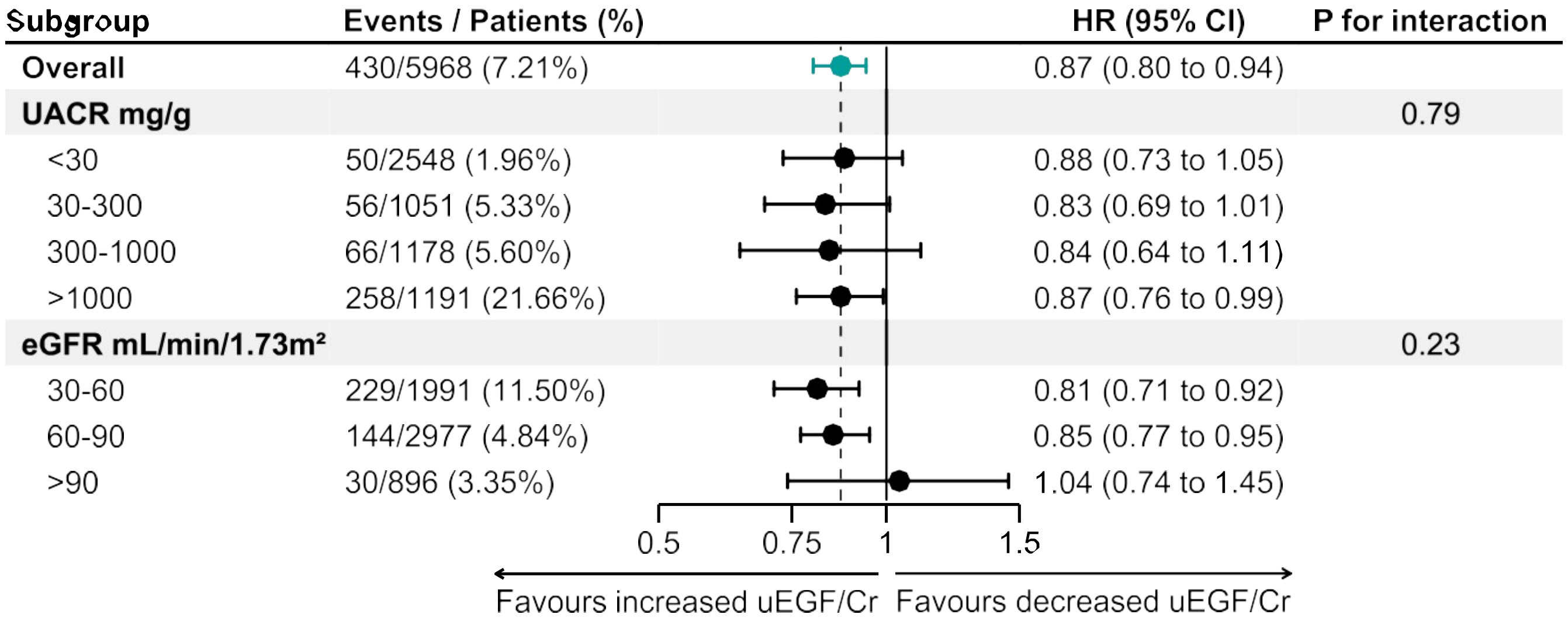


*Association is shown between uEGF/Cr with the kidney outcome by UACR and eGFR subgroups.*

*HRs are expressed per 2-fold higher uEGF/Cr and adjusted for age, sex, race, trial, randomized treatment, history of CVD, HbA1c, smoking, systolic BP, BMI, eGFR (not for eGFR subgroups), and log-transformed UACR (not for UACR subgroups).*

*Abbreviations: CI: confidence interval; HR: hazard ratio; uEGF/Cr: urinary epidermal growth factor-creatinine ratio; CVD: cardiovascular disease; HbA1c: hemoglobin $A_{1c}$; BP: blood pressure; BMI: body mass index; eGFR: estimated glomerular filtration rate; UACR: urine albumin-creatinine ratio;*

**Supplemental Figure 2:** Association of change in uEGF/Cr from baseline to year 1 with kidney outcome by eGFR and UACR subgroups in

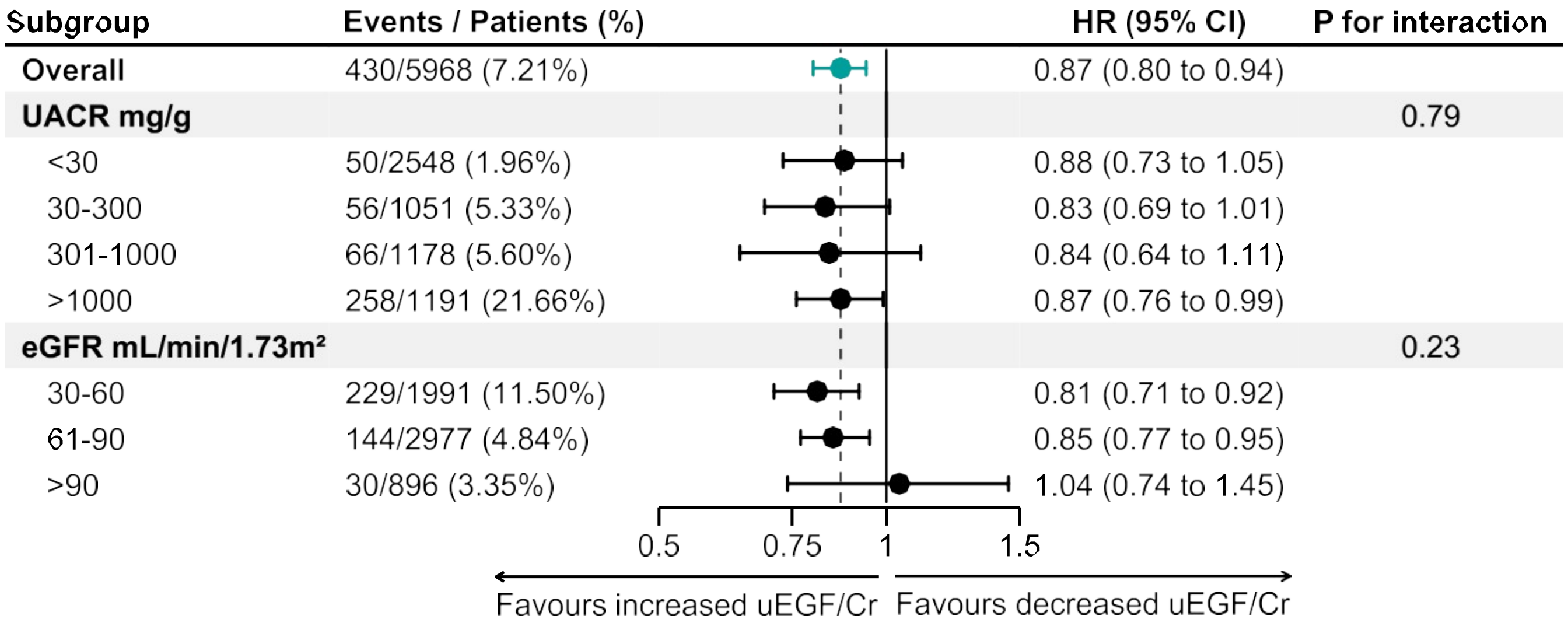


*Association is shown between change in uEGF/Cr from baseline to year 1 with the kidney outcome by UACR and eGFR subgroups. HRs are expressed per 2-fold increase in uEGF/Cr and adjusted for baseline log uEGF/Cr, age, sex, randomized treatment, trial, history of CVD, HbA1c, smoking, systolic BP, BMI, eGFR (not for eGFR subgroups), and log-transformed UACR (not for UACR subgroups) and change in eGFR (not for eGFR subgroups), UACR (not for UACR subgroups), systolic BP, and HbA1c from baseline to year 1.*

*Abbreviations: CI: confidence interval; HR: hazard ratio; uEGF/Cr: urinary epidermal growth factor-creatinine ratio; CVD: cardiovascular disease; HbA1c: hemoglobin $A_{1c}$; BP: blood pressure; BMI: body mass index; eGFR: estimated glomerular filtration rate; UACR: urine albumin-creatinine ratio;*

**Supplemental Figure 3:** Associations of change in uEGF/Cr from baseline to year 1 with kidney outcomes in DAPA-CKD participants with and without type 2 the distribution of uEGF/Cr changes.

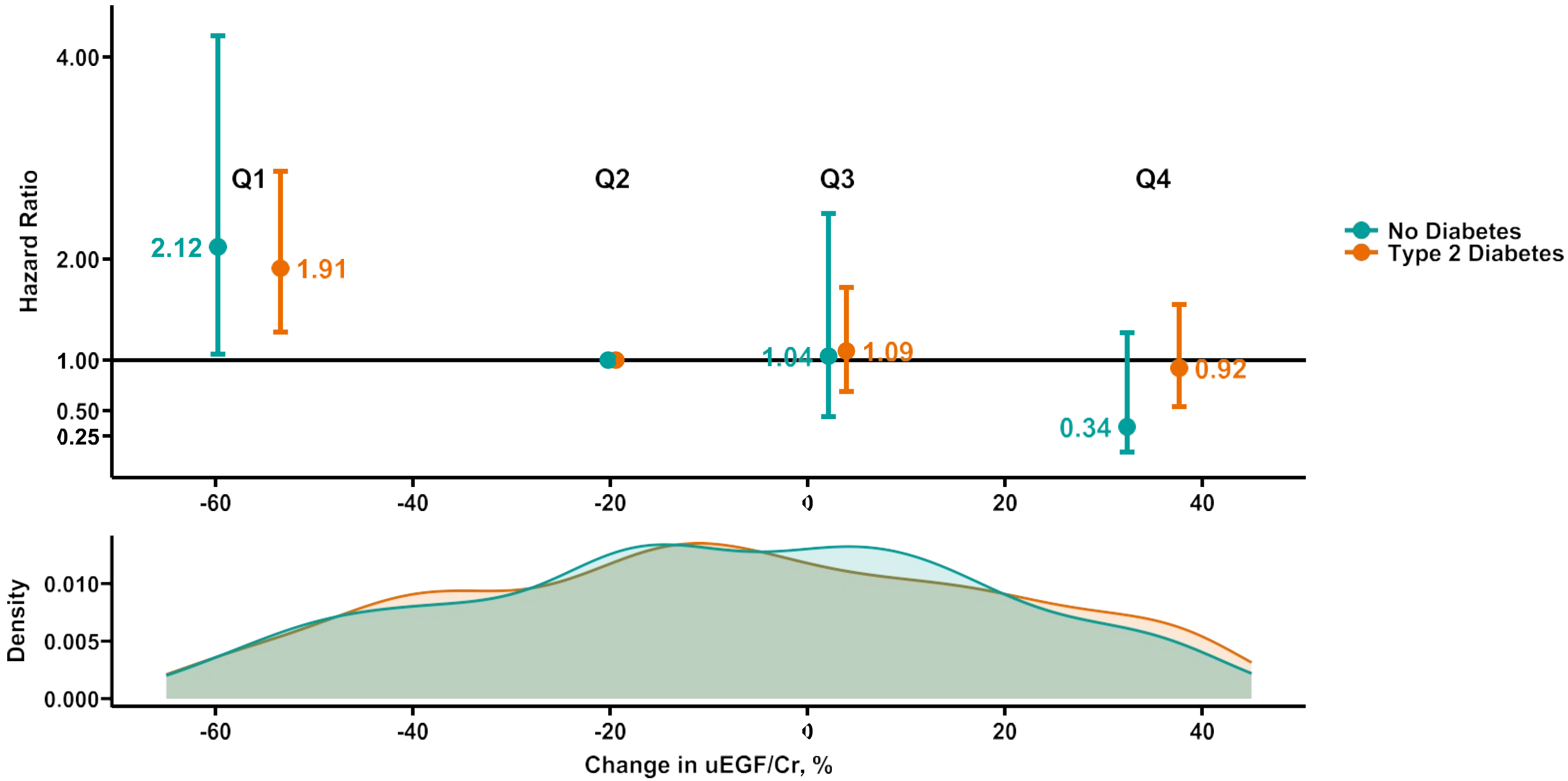


*Association is shown between change in uEGF/Cr from baseline to year 1 and the kidney outcome when uEGF/Cr change is modeled as a categorical (quartiles) variable, stratified by diabetes status. Dots represent HRs for each quartile at the median change, bars indicate 95% CI.*

*Model is adjusted for the following covariates: baseline log uEGF/Cr, age, sex, race, randomized treatment, history of CVD, HbA1c, systolic BP, BMI, eGFR, and log UACR at baseline and change in eGFR, UACR, systolic BP, and HbA1c from baseline to year 1. Abbreviations: CI: confidence interval; HR: hazard ratio; uEGF/Cr: urinary epidermal growth factor-creatinine ratio; CVD: cardiovascular disease; HbA1c: hemoglobin A1c; BP: blood pressure; BMI: body mass index; eGFR: estimated glomerular filtration rate; UACR: urine albumin-creatinine ratio*
